# Feature selection and aggregation for antibiotic resistance GWAS in *Mycobacterium tuberculosis*: a comparative study

**DOI:** 10.3389/fmicb.2025.1586476

**Published:** 2025-06-18

**Authors:** Kirill Reshetnikov, Daria Bykova, Konstantin Kuleshov, Konstantin Chukreev, Egor Guguchkin, Alexey Neverov, Gennady Fedonin

**Affiliations:** ^1^Central Research Institute of Epidemiology, Moscow, Russia; ^2^Faculty of Bioengineering and Bioinformatics, Moscow State University, Moscow, Russia; ^3^Federal State Budget Scientific Institution “Federal Scientific Center VIEV”, Moscow, Russia; ^4^Ufa Federal Research Center of the Russian Academy of Sciences, Ufa, Russia; ^5^Faculty of Computer Science/Institute of Artificial Intelligence and Digital Sciences/International Laboratory for Statistical and Computational Genomics, HSE University, Moscow, Russia

**Keywords:** *Mycobacterium tuberculosis*, antimicrobial drug resistance, feature selection, machine learning, PFAM domains

## Abstract

**Introduction:**

Drug resistance (DR) of pathogens remains a global healthcare concern. In contrast to other bacteria, acquiring mutations in the core genome is the main mechanism of drug resistance for *Mycobacterium tuberculosis* (MTB). For some antibiotics, the resistance of a particular isolate can be reliably predicted by identifying specific mutations, while for other antibiotics the knowledge of resistance mechanisms is limited. Statistical machine learning (ML) methods are used to infer new genes implicated in drug resistance leveraging large collections of isolates with known whole-genome sequences and phenotypic states for different drugs. However, high correlations between the phenotypic states for commonly used drugs complicate the inference of true associations of mutations with drug phenotypes by ML approaches.

**Methods:**

Recently, several new methods have been developed to select a small subset of reliable predictors of the dependent variable, which may help reduce the number of spurious associations identified. In this study, we evaluated several such methods, namely, logistic regression with different regularization penalty functions, a recently introduced algorithm for solving the best-subset selection problem (ABESS) and “Hungry, Hungry SNPos” (HHS) a heuristic algorithm specifically developed to identify resistance-associated genetic variants in the presence of resistance co-occurrence. We assessed their ability to select known causal mutations for resistance to a specific drug while avoiding the selection of mutations in genes associated with resistance to other drugs, thus we compared selected ML models for their applicability for MTB genome wide association studies.

**Results and discussion:**

In our analysis, ABESS significantly outperformed the other methods, selecting more relevant sets of mutations. Additionally, we demonstrated that aggregating rare mutations within protein-coding genes into markers indicative of changes in PFAM domains improved prediction quality, and these markers were predominantly selected by ABESS, suggesting their high informativeness. However, ABESS yielded lower prediction accuracy compared to logistic regression methods with regularization.

## Introduction

### Tuberculosis chemotherapy and the problem of drug resistance

Tuberculosis (TB), caused by *Mycobacterium tuberculosis* (MTB), remains a leading cause of death from a single infectious agent, despite a decrease in annual TB mortality rates in recent years (World Health Organisation, 2020). TB is a curable disease, typically treated effectively with a combination of drugs. Until 2020, when the WHO treatment guidelines were significantly updated, the two basic schemes of TB therapy had been applied (Jnawali and Ryoo, 2013). Naive patients were effectively treated with a combination of first-line drugs including rifampicin (RIF), isoniazid (INH), pyrazinamide (PZA), ethambutol (EMB). Streptomycin (STR) was used for TB treatment since 1945, initially as monotherapy and later as part of the first-line regimen after other first-line drugs were endorsed. Due to the high prevalence of resistance and serious adverse effects, it has been used in addition to other first-line drugs since 1991 in cases of treatment failure. Today, streptomycin is still recommended for restrictive use as a substitute for amikacin (Rocha et al., 2021; Singh et al., 2015). Relapse cases were treated with second-line drugs such as fluoroquinolones (ofloxacin (OFX), levofloxacin (LEV), moxifloxacin (MOX) and ciprofloxacin (CIP)) and injectable drugs (kanamycin (KAN), amikacin (AMK) and capreomycin (CAP)). In 2020, the WHO endorsed several new drugs for treating MDR-TB, including bedaquiline, linezolid, clofazimine, cycloserine and terizidone. At the same time, KAN and CAP were excluded from use, and the priority of drugs for inclusion in combination therapies was revised (Migliori et al., 2020). Recent recommendations provide the highest priority for fluoroquinolones, bedaquiline and linezolid as the second-line drugs for TB treatment (World Health Organisation, 2020). Other second-line anti-TB drugs, such as ethionamide (ETH)/prothionamide (PTH), cycloserine (CS), P-aminosalicylic acid (PAS), are considered to be less effective. Recently, there has been growing concern over the increasing fraction of multidrug resistant (MDR) isolates, which are resistant to the most potent anti-TB agents rifampicin (RIF) and isoniazid (INH), as well as extensively drug-resistant (XDR) isolates, which were defined as MDR with additional resistance to any of aminoglycosides and fluoroquinolones until 2021 (World Health Organisation, 2020). The definition of XDR-TB was updated in 2021 in alignment with changes in treatment regimens (WHO Announces Updated Definitions of Extensively Drug-Resistant Tuberculosis, 2024). In this study, we used former definitions of first-and second-line drug classes and the XDR-TB (Jnawali and Ryoo, 2013).

Mutations in genes targeted by drugs are a main mechanism of drug resistance in MTB (Trauner et al., 2017). Additionally, if MTB develops resistance to one drug, it often acquires resistance to other drugs more easily (Moura et al., 2017).

Several laboratory methods exist for detecting MTB DR. The ‘gold standard’ involves measuring the minimal concentrations of substances that inhibit bacterial growth to assess resistance/susceptibility levels (Andrews, 2001). However, phenotypic tests are time-consuming due to MTB’s slow growth, expensive, and can only be performed in laboratories with high biosafety levels. As a result, genotypic tests have gradually been replacing them.

### Interpretation of phenotypic effects of mutations

Most methods for predicting MTB phenotype from genotype utilize the direct association (DA) approach based on the interpretation of detected mutations as benign or causal (Chen et al., 2019). Prior knowledge of genes likely involved in drug action has facilitated the identification of mutations that confer drug resistance. A pioneering DA approach was developed by Walker et al. (2015), followed by the creation of other catalogs of resistance-causing mutations based on thorough literature reviews (Allix-Béguec et al., 2018; Domínguez et al., 2016; Miotto et al., 2017), machine learning methods (Farhat et al., 2016) and comparative genomics (Ghosh and Saha, 2020). Recently, WHO standardized methodology for compiling a catalog of mutations best explaining drug phenotypes (Walker et al., 2022). We have compiled a list of known or proposed MTB genes with mutations associated with resistance to RIF, INH, PZA, EMB, STR, AMK, KAN, CAP, MOX, CIP, OFX, ETH and PTH in Supplementary Table S1.

### Looking for new mutations conferring drug resistance

Direct association models perform well for some drugs with well-known mechanisms of action, e.g., rifampicin and isoniazid. However, for other drugs, e.g., pyrazinamide, ethambutol, fluoroquinolones, mutations in known genes may not fully explain phenotypes (Chen et al., 2019; Schleusener et al., 2017). The number of potential loci contributing to drug resistance is large (Farhat et al., 2019). For instance, in experiments with *E. coli*, high levels of resistance can result from the modulation of expression of a large number of functionally diverse genes (Palmer et al., 2018). Consequently, the discovery of new causal mutations remains an ongoing challenge.

Many variations of genome-wide association studies (GWAS) were used to identify new genome loci associated with MTB drug resistance, analyzing datasets that include nearly complete-genome genotypes along with characterized drug phenotypes. Some methods are based on the detection of sites with recurrent mutations in phylogenetically unrelated strains (Zhang et al., 2013; Farhat et al., 2013; Coll et al., 2018; Hicks et al., 2018; Hicks et al., 2019), others utilize machine learning (ML) approaches such as k nearest neighbors (Sergeev et al., 2019), regularized logistic regression (Sergeev et al., 2019; Niehaus et al., 2014; Lees et al., 2020), support vector machine (Niehaus et al., 2014; Yang et al., 2017; Kavvas et al., 2018), gradient-boosted trees (Sergeev et al., 2019; Deelder et al., 2019), random forest (Sergeev et al., 2019; Yang et al., 2017), mixture models (Yang et al., 2017) and linear mixed models (LMM) that account for the clonal structure of MTB population (Farhat et al., 2019; Coll et al., 2018; Earle et al., 2016). New methods are constantly being developed, for example, the one based on boolean compressed sensing (Zabeti et al., 2021).

### Correlations between phenotypic states of commonly used drugs complicate interpretation of GWAS results

Although a few newly identified loci have been experimentally confirmed as causal (Palmer et al., 2018; Farhat et al., 2013; Coll et al., 2018), many others require verification and may be spuriously associated. Compared to the DA approach, the accuracy of drug phenotype prediction has been significantly improved by the application of ML to mutations occurring in known drug-associated genes (Chen et al., 2019; Farhat et al., 2016; Kavvas et al., 2018; Yang et al., 2018; Yang et al., 2019; Kouchaki et al., 2019). The improvement is partly achieved due to the high correlation between phenotypic states for drugs usually prescribed in combination (Yang et al., 2018; Kouchaki et al., 2019). The presence of a specific mutation that causes resistance to one drug informs a model about high probability of resistance to other drugs. For example, the *katG* S315T mutation conferring resistance to INH typically arises before acquisition of RIF resistance (Cohen et al., 2015; Manson et al., 2017), making it a good predictor for MDR, especially when RIF resistance is caused by rare *rpoB* mutations (van et al., 2001; Hazbón et al., 2006). Such correlations can pose challenges for ML models trained on datasets with properties different from the real-life datasets (Kouchaki et al., 2019). Additionally, models based on resistance co-occurrence are unable to capture rarer causal mutations because covariates would mask weak signals (Yang et al., 2018). Finally, such models may not produce new insights into the mechanisms of drug resistance.

### Dealing with rare mutations

Alongside resistance co-occurrence, one of the major problems in GWAS is the ‘curse of dimensionality’, driven by the high dimensionality of ML models and the modest sizes of training datasets. Most mutations in the MTB genome are rare, occurring in only one or two isolates in the datasets comprising tens of thousands of sequences (Stritt and Gagneux, 2023). As a result, their phenotypic effects could not be reliably estimated. Aggregation of mutations with the subsequent exclusion of rare mutations is a way of dimensionality reduction. It is typically done by constructing features indicating the presence of mutations in specific genes or regulatory regions (Chen et al., 2019). For *pncA*, which carries multiple mutations impairing its function, Karmakar et al. (2020) endorsed a method of mutation aggregation based on the prediction of their impacts. They employed a rather complicated model requiring a set of mutations with known effects for training, which makes it impossible to generalize for any MTB protein. Aggregated features reduce dimensionality without loss of interpretability and allow for the accounting for mutations not present in the training set. In this study, we used HMM models from the protein families database (PFAM) for aggregation as a mechanism to predict the impacts of mutations on protein functions.

### Overview of the feature selection methods

In ML, feature selection is a process of selecting a subset of the most relevant variables (features) for use in a predictive model. In our study, features are binary variables, each representing the presence or absence of a specific mutation at a particular site in the genome. Additionally, we include features for complex events, such as “broken gene” and “PFAM domain change,” which may aggregate multiple mutations and are also encoded as binary variables. We use linear models to predict binary phenotypic states, i.e., resistance to a specific drug, based on genomic features. We compared multiple feature selection methods in terms of both their prediction accuracy and the biological relevance of the identified associations between genes and drugs. These methods included logistic regression with various regularization techniques: lasso (L1) (Tibshirani, 1996), minimax concave penalty (MCP) (Zhang, 2010), smoothly clipped absolute deviation (SCAD) (Fan and Li, 2001) and elastic net (Zou and Hastie, 2005). We also employed an algorithm for the best-subset selection (ABESS), which searches for the smallest subset of features that best explain drug phenotypes (Zhu et al., 2020). Additionally, we used a heuristic algorithm specially designed for GWAS in datasets with highly correlated features – “Hungry, Hungry SNPos” (HHS) (Libiseller-Egger et al., 2020). We assume that our data exhibit high heterogeneity in the strength of mutation effects, i.e., some mutations have strong effects on a phenotypic state, while others have weak effects. We also assume that the true model is sparse, with only few features having nonzero effects relative to the total number of features. All of these ML models are well-suited for feature selection in settings with sparse, heterogeneous effects and highly correlated features (Roy et al., 2023).

The logistic regression model searches for additive coefficients *β* of genomic variations to maximize the likelihood of the dataset. Wald and likelihood statistics allow estimating statistical significance of coefficients *β* (Richardson, 2011). Likelihood maximization tends to assign nonzero weights to all features, underestimating the impact of causal features and overfitting the noise. Also, due to correlations with benign features, the causal ones may be overlooked during feature selection. Lasso regularization (Tibshirani, 1996) adds a linear regularization term to the likelihood function, penalizing large coefficients, which is also known as L1 norm regularization. As a result, it selects only one feature from a subset of highly correlated ones. Ridge regression (Hoerl and Kennard, 1970a; Hoerl and Kennard, 1970b) penalizes the squared $\underline{\beta }$ coefficients (L2 norm), while elastic net combines both L1 and L2 penalties. Lasso has been shown to outperform unregularized stepwise feature selection in both prediction quality and the proportion of causal features in the selected set in simulated data (Tibshirani, 1996; Grogan and Elashoff, 2016). In simulated bacterial GWAS, lasso performs better or similarly to linear mixed models (LMMs), even in the presence of high linkage disequilibrium (LD) and strong population structure (Saber and Shapiro, 2020). Elastic net has earlier been shown to outperform lasso in prediction quality (Lees et al., 2020).

Penalizing large coefficients comes at a price: large true coefficients may also be penalized. While regularization techniques each have their drawbacks and limitations, new methods are constantly being developed. Fan and Li introduced SCAD regularization which applies a nonlinear decreasing penalty to large coefficients (Fan and Li, 2001). A similar effect is achieved by MCP regularization (Zhang, 2010). In feature selection, MCP and SCAD outperformed lasso on all types of simulated data, except for non-sparse data with a low signal-to-noise ratio, in which case the elastic net was the leader (Kumar et al., 2021).

Some methods penalize the number of nonzero coefficients (L0 norm regularization), which makes the optimization function non-differentiable and requires enumerating feature subsets to identify the best one. ABESS efficiently implements enumeration and optimizes various information criteria (AIC, BIC, cross-validation, and an original asymptotic criterion - SIC) to find the best subset (Zhu et al., 2020). For each feature set size, starting from one up to the user-defined maximum, the best subset of features is constructed, and the final subset is selected based on the chosen information criterion. The best subset of a given size is constructed by first selecting features most strongly correlated with the target variable (the binary phenotypic state, in our case). The solution is iteratively improved by scoring all features based on the content of the currently selected set and swapping the least informative features with the most informative ones. This procedure is shown to converge in a finite number of steps (Zhu et al., 2020). A thorough theoretical analysis of ABESS is provided in the original paper, where the authors study the sufficient conditions for the algorithm’s correctness and prove that, under such conditions, its running time is polynomial. Like LASSO, SCAD, and MCP, ABESS can identify the correct model if feature correlations are not too high, the true number of causal features is small, and the true coefficients are sufficiently large, while the data is not too sparse. All the methods have difficulties when there are many features with small effects (Zhu et al., 2020).

ABESS outperformed SCAD, MCP, and Lasso regularization on simulated data in the original study (Tomasicchio et al., 2016). However, other benchmarks comparing these methods on different simulated datasets have yielded inconsistent results, suggesting that their performance depends on the underlying data structure (Hanke et al., 2020; Roy et al., 2023).

In addition to well-known and theoretically grounded approaches described above, new heuristic methods are being developed for GWAS to address feature correlations by taking the population structure into account (Libiseller-Egger et al., 2020). The HHS algorithm begins by assigning a score to each SNP, reflecting the correlation with phenotype while adjusting for the population structure. The score can be modified to account for class imbalance in the phenotype and allele frequencies. Similar to ABESS, the currently selected set of features (all having positive scores) is iteratively optimized by decreasing the normalized scores of mutually correlated features until convergence (Libiseller-Egger et al., 2020).

We benchmarked these methods on a large dataset and evaluated their ability to select biologically relevant genes and associate them with the proper drugs, while being trained on samples exhibiting resistance co-occurrence. Based on our benchmarking results, we propose a two step feature selection procedure utilizing ABESS, which emerged as the top performer in our evaluation. This procedure aims to uncover new associations that may have been missed during the initial selection step.

## Methods

Supplementary Figure S1 summarizes the main steps of our study.

### Dataset preparation

The dataset comprises phenotypically characterized whole-genome sequences of *M. tuberculosis* gathered from multiple studies (Walker et al., 2015; Farhat et al., 2019; Zhang et al., 2013; Farhat et al., 2013; Coll et al., 2018; Yang et al., 2017; Kouchaki et al., 2019; Casali et al., 2014; Pankhurst et al., 2016; Johnsen et al., 2019). Illumina reads were trimmed and mapped to the H37Rv reference genome (AL123456.3). Variants were called with GATK HaplotypeCaller and multiple filtering steps were applied.

For each isolate, the nucleotide sequence of every protein-coding gene was translated, the corresponding amino acid sequence was aligned to the reference protein sequence carefully accounting for potential frameshifts and start or stop codon loss. Genes with mutations caused the resulting protein to be 50% shorter or 30% longer than the reference were classified as ‘broken’, and mutations within these genes were excluded from consideration. Finally, we obtained a list of mutations relative to the reference genome including mutations in noncoding regions, missense mutations, loss-of-function mutations, and aggregated features (see below). Synonymous mutations were excluded from the analysis.

The final dataset was divided into subsets containing only isolates with known phenotypes for each antibiotic. Aggregation of rare mutations and machine learning were performed separately for each subset. For each drug, the corresponding subset was randomly split into five non-overlapping subsets (folds). Each fold was used as the testing set one at a time, while the union of the remaining folds served as the training set. Below we refer to every such partitioning of the dataset into the training and the testing sets “the dataset split” (see “Dataset and raw data processing” in Supplementary Methods and Supplementary Figures S1, S2 for details).

### Aggregation of mutations

We examined three types of aggregated features: (i) an indicator of the presence of any mutation in a gene (“gene aggregation” feature), (ii) an indicator that a gene is broken, i.e., it has a loss-of-function mutation (“broken gene” feature), and (iii) features indicating that a function of a PFAM domain was likely affected by mutations (“PFAM domain” features).

If there is any mutation in a gene, the “gene aggregation” feature is assigned a value of “1,” and “0” otherwise. “Broken gene” feature is assigned a value “1” if the length of the encoding amino acid sequence is sufficiently different from the length of the gene product in the reference genome, otherwise it is assigned a value “0” (see the previous section).

To generate PFAM domain features we used pre-trained models available for 3,369 *M. tuberculosis* genes in the PFAM database.^1^ Using these models for amino acid sequences of domains, we computed scores representing the probabilities that domain sequences were generated by corresponding HMM models. We then converted these scores into binary variables by selecting a threshold for each domain and for each drug separately, optimizing the separation of resistant and susceptible sequences in the training set of each dataset split. If a domain score exceeded the threshold, the function of the domain was considered to be affected by mutations and the corresponding binary variable was assigned a value of “1” and a value “0” otherwise (see “PFAM domains based feature aggregation” in Supplementary Methods).

### Estimating the impact of aggregation

To measure the effect of aggregation, we evaluated results of classification of isolates into resistant and susceptible phenotypic states with logistic regression with L1 regularization using different feature set combinations. The basic feature set included mutations only, comprising SNPs and Indels, without aggregation. Three other feature sets contained both aggregated features and mutations, excluding rare mutations occurring <3 times in the training set: (1) SNPs and Indels with PFAM domain features; (2) SNPs and Indels with “broken gene” features; (3) SNPs and Indels with “gene aggregation” features. In these three sets and in other analyses described below, rare mutations were filtered out after the generation of aggregated features.

We computed ROC AUC and F1 scores using 5-fold cross-validation. Logistic regression was trained on the training sets of dataset splits, and the scores computed on testing sets were averaged. To assess the statistical significance of the differences in scores between the models trained on different feature sets, we repeated this procedure 100 times. We then calculated the Wilcoxon paired rank test between two vectors of length 100 of corresponding scores. The null model assumes that there is no difference between the performance of logistic regression on different feature sets and the difference of scores obtained on 5-fold cross-validation is explained only by chance. We considered differences to be significant if the *p*-value, with Bonferroni correction to the number of drugs, was lower than 5%.

The scheme of the comparison of feature aggregation strategies is presented in Supplementary Figure S2.

### Feature selection and model quality evaluation

We evaluated the performances of several methods – regularized logistic regression (L1, MCP and SCAD), elastic net, HHS and ABESS – using 5-fold cross-validation for each drug separately. After the training of each method on each of the five training sets, we obtained a list of features with non-zero coefficients. For each feature, we computed the number of times it was selected by each method.

We defined a feature as ‘majorly selected’ if it was selected in three or more dataset splits. For each such feature, we computed Fisher’s exact test p-value for its association with phenotypic states on the testing set of the first dataset split. To estimate the prediction accuracy of the subsets of selected features, we trained the ordinary logistic regression using these features, and computed ROC AUC, sensitivity, specificity, NPV and PPV.

To search for additional features associated with drug phenotypes, we performed a second iteration of ABESS on the subset of data unexplained by the majorly selected features from the first iteration. For the second iteration of ABESS, we removed resistant isolates correctly classified on the previous iteration and some randomly selected susceptible isolates to maintain class balance. All features majorly selected for at least one drug with positive coefficients in the first iteration were completely removed to prompt the method to search for the new associations. After training of ABESS, the feature selection process was repeated. The same procedure was performed for HHS (see Supplementary Methods for details).

### Accounting for the population structure

It is widely accepted that due to clonal evolution of bacteria, certain multicomponent genetic features associated with resistance may be shared by groups of strains with common ancestry. These groups may comprise distinct clades on the phylogenetic tree, which may be associated with geographic regions or specific MTB genetic lineages. Therefore, genetic markers of such clades could spuriously be associated with drug resistance or susceptibility by any statistical learning method.

To investigate if there are any subsets of phylogenetically related isolates in our dataset where shared ancestry serves as a marker of resistance, we constructed a maximum likelihood phylogenetic tree for all isolates included in our study (see Supplementary Methods for details). For each drug, we pruned all isolates lacking phenotype information and obtained the corresponding subtree. We then applied TreeBreaker (Ansari and Didelot, 2016) to each subtree to infer clades with a prevalence of resistant isolates significantly different from parental clades. Branches of each subtree having posterior probabilities of prevalence switches larger than 0.5 were used as structural binary features, taking a value of one for all descendent isolates and zero for all other isolates.

Geographic locations of isolates were obtained from NCBI BioSample records. If location data were missing in a BioSample record, the location of an isolate was assigned based on text descriptions in the corresponding BioProject record, if possible. We used the information about a country, where an isolate had been sampled, for further analysis. To test if the distribution of isolates belonging to each clade identified by TreeBreaker by countries was significantly different from the distribution of all isolates, we computed a sum of squares of differences of isolate frequencies within country categories belonging to the TreeBreaker clades and frequencies of isolates in the entire tree. We computed *p*-values for this statistic by multiple permutations of location labels of isolates. Bonferroni correction for the number of applied tests for all subtrees together was used for the p-values. We considered significant p-values below the Bonferroni corrected 5% threshold.

Isolates were assigned to the MTB genetic lineages based on the catalog of the TB-Profiler lineage-defining mutations (Bosch et al., 2021). To test if the distribution of isolates belonging to each clade identified by the TreeBreaker by lineages was significantly different from the distribution by lineages of all isolates, we applied the similar procedure as for the geographic location test.

Isolates that had no location or lineage information were ignored in the corresponding tests (see Supplementary Methods for details).

## Results

### Most mutations are rare

Short reads and phenotypes for 13 drugs were obtained from publicly available sources (Supplementary Table S2) (Walker et al., 2015; Farhat et al., 2019; Zhang et al., 2013; Farhat et al., 2013; Coll et al., 2018; Yang et al., 2017; Kouchaki et al., 2019; Casali et al., 2014; Pankhurst et al., 2016; Johnsen et al., 2019). After subsequent data preprocessing, our dataset comprised 12,333 whole-genome sequences for which binary phenotype (resistance or susceptibility) for at least one of 13 antibiotics was known.

For all antibiotics, the number of mutations significantly exceeded the number of isolates. Moreover, most mutations (>67%) were rare: they appeared in the dataset in three or less isolates (Table 1).

**Table 1:** The descriptive statistics of the dataset.

Drug name	Classification by the treatment line	Drug group	Number of isolates with known phenotype	Resistance fraction	Number of mutations	Fraction of indels among all mutations	Fraction of rare mutations
Rifampicin	First line		11,913	24.2%	301,227	3.6%	77.1%
Isoniazid			11,828	29.5%	300,368	3.6%	77.1%
Pyrazinamide			9,329	12.0%	261,081	3.6%	77.6%
Ethambutol			10,346	14.9%	287,143	3.6%	77.9%
Streptomycin		Aminoglycosides	4,805	35.6%	149,347	3.8%	77.6%
Kanamycin	Second line		1,209	28.7%	45,512	4.6%	74.8%
Amikacin			1,878	21.7%	47,794	4.5%	72.8%
Capreomycin			1,959	21.0%	54,308	4.6%	75.2%
Ofloxacin		Fluoroquinolones	1,974	24.1%	55,591	4.5%	76.4%
Moxifloxacin			1,223	21.4%	36,115	4.8%	74.1%
Ciprofloxacin			471	22.3%	30,313	4.2%	74.8%
Ethionamide		Thiocarbamide derivative	700	40.3%	27,284	4.5%	71.0%
Prothionamide			442	40.7%	8,231	5.1%	67.9%

We define a mutation as rare if it occurs in fewer than three genotypes in the dataset.

### The phenotypic states of most drugs are coherent

**Figure 1:**
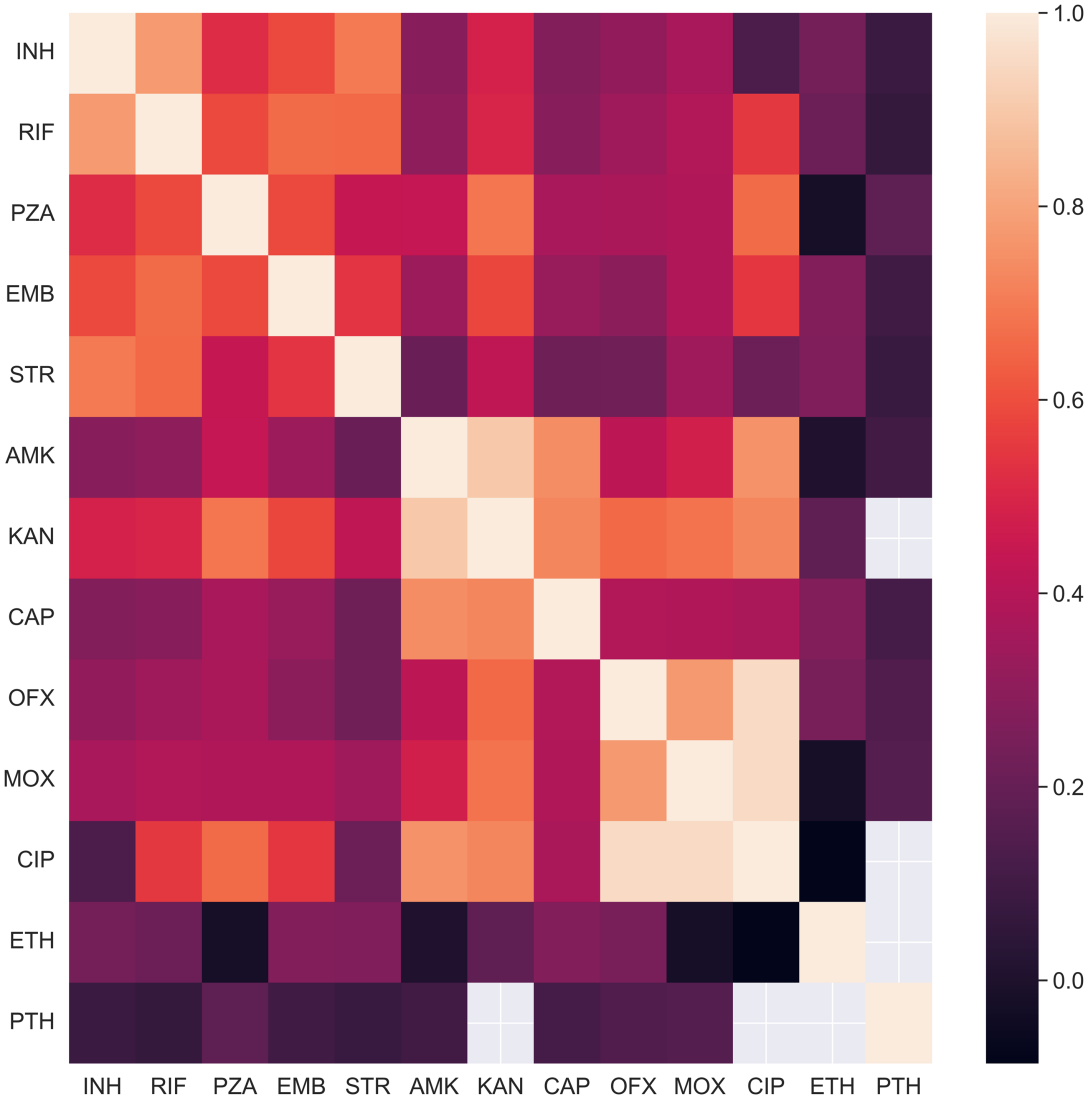
Concordance of drug resistance acquisition between co-administered drugs and drugs within common pharmacological groups. The heatmap displays the Pearson correlation between phenotypic states for drug pairs. Correlations were calculated using only isolates with known phenotypic states for both drugs in each pair.

To illustrate and quantitatively characterize the coherence of phenotypic states, we computed Pearson’s correlations for all pairs of drugs. The correlation was computed on the isolates phenotypically characterized for both drugs in a pair. The resulting heatmap is presented in Figure 1. Many pairs of phenotypes turned out to be strongly correlated. High correlations are expected for drugs from one pharmacological group such as aminoglycosides (amikacin, kanamycin, streptomycin, capreomycin) and fluoroquinolones (moxifloxacin, ofloxacin, and ciprofloxacin). Aminoglycosides share three known genes associated with resistance (*rrs*, *tlyA*, *gidB*) and fluoroquinolones share two (*gyrA*, *gyrB*) (Table 1). Another cause of high correlation is joint antibiotic treatment, which is probably the case for rifampicin and isoniazid, as well as for all pairs of the first-line drugs.

### Aggregation of mutations improves phenotype prediction quality

After exploring the dataset, we focused on creating an effective representation of genotypes to evaluate feature selection models. Due to the predominance of rare mutations and the substantially higher total number of mutations compared to the number of isolates in the dataset for some drugs, we generated the following aggregated features: “broken genes,” “gene aggregation” and “PFAM domains” (see “Methods”). We then created three feature sets, each containing one of the aggregated features along with mutations, excluding rare mutations occurring in fewer than three isolates. To compare the feature sets with aggregated features to the initial feature set, which included all mutations without aggregation, we evaluated the classification accuracies of logistic regression models with L1 regularization, trained on these sets for the binary drug phenotype prediction task.

**Figure 2:**
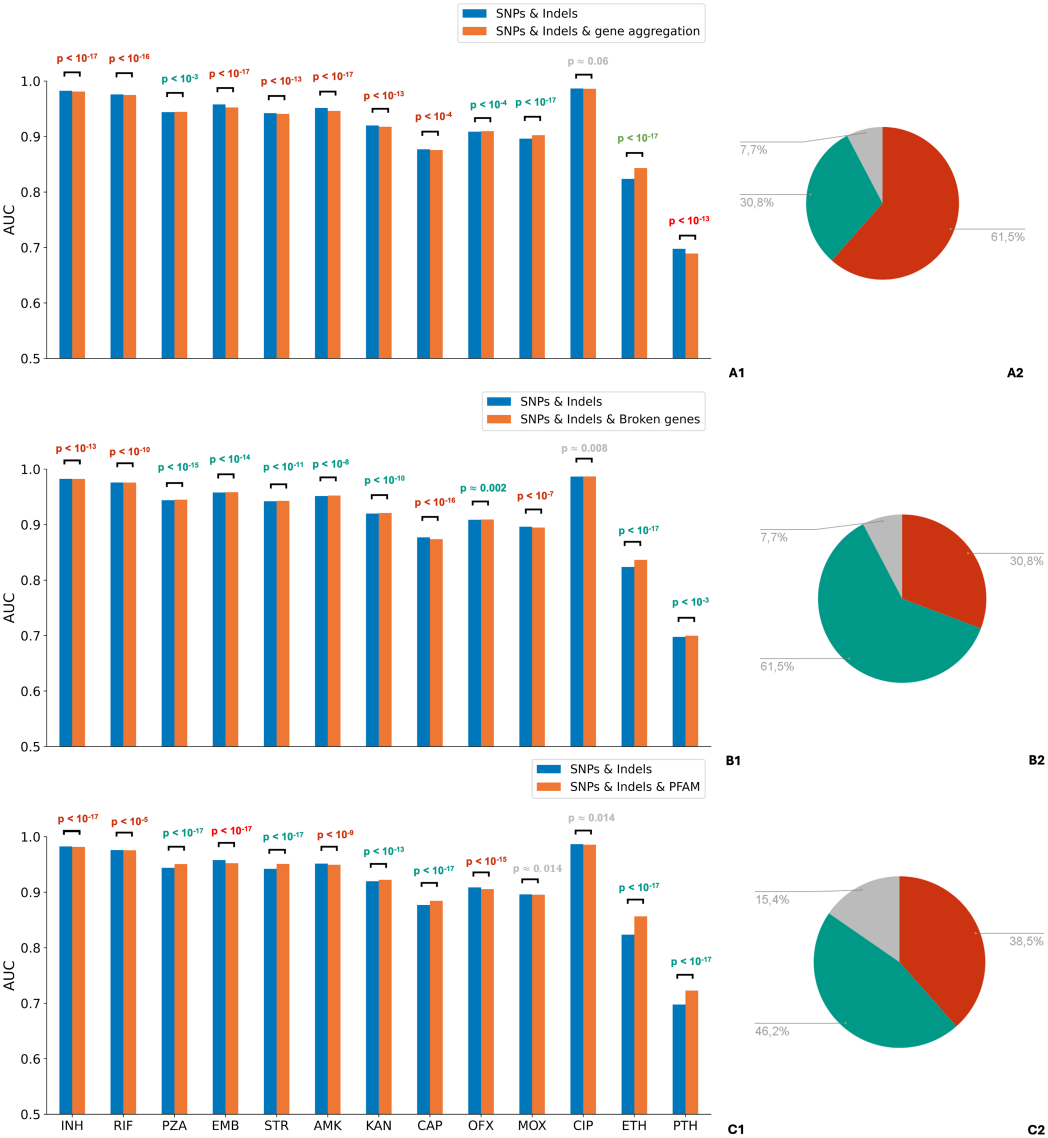
Comparison of drug resistance prediction accuracies using the original mutation feature set and feature sets with various aggregations of rare features. Aggregation of loss-of-function mutations and mutations occurring in PFAM domains likely affecting protein functions, improves, rather than compromises, accuracy for most drugs. Panels **A1–C1**: comparison of mean ROC AUC scores of logistic regression evaluated on different feature sets across drug groups: **(A1)** “SNPs and Indels” vs “SNPs and Indels and gene aggregation excluding rare mutations”; **(B1)** “SNPs and Indels” vs “SNPs and Indels and broken genes excluding rare mutations”; **(С1)** “SNPs and Indels” vs “SNPs and Indels and PFAM domains excluding rare mutations.” The Wilcoxon signed-rank test was used to evaluate statistical significance. *p*-values for drug groups that demonstrated a statistically significant increase in AUC due to aggregation are colored in teal, while those showing a significant decrease are colored in red. Panels **A2–C2**: the pie charts display the percentage of drugs for which models trained on datasets with feature aggregation outperform (shown in teal), underperform (shown in red), or have no effect on the score (shown in gray).

The feature sets containing aggregated features demonstrated significantly higher phenotypic state prediction quality for some drugs compared to feature sets with no aggregation (Figure 2 and Supplementary Tables S3–S8). Although the differences in ROC AUC values were moderate, they often exhibited large statistical significance due to small variances across all feature sets (Supplementary Table S9). Feature sets incorporating PFAM domain features yielded higher AUC scores for all drug groups except fluoroquinolones and for six drugs individually. However, for four drugs, domain features impaired the classification accuracies, and for three others, AUC scores differed insignificantly (Supplementary Table S3). Models trained on the dataset including “broken genes” features outperformed the baseline models in pairwise comparisons for 8 out of 13 drugs, for four drugs they decreased AUCs (Supplementary Table S4). “Gene aggregation” feature was superior only for 5 out of 13 drugs, while for 7 drugs the result was the opposite (Supplementary Table S5). Results for the F1 score were generally consistent with ROC AUC results (Supplementary Tables S6–S8). We also compared PFAM domain features with gene aggregation. PFAM domain-based aggregation outperformed gene aggregation for most drugs in terms of both ROC AUC and F1 score (Supplementary Tables S10, S11). Based on these findings, we retained PFAM domain features and “broken genes” features in all further experiments.

### Comparison of feature selection algorithms

In this study, we explored six methods for the selection of causal DR mutations: logistic regression with L1, MCP, and SCAD regularization, elastic net, HHS, and ABESS. Feature selection models were trained on the training parts of five dataset splits for drug resistance prediction tasks (separately for each drug). The mutations selected in at least one dataset split by each method are listed in Supplementary Tables S12–S17, along with the number of dataset splits in which each mutation was chosen. For each method, we focused on features selected in at least three splits and evaluated their predictive power and, importantly, the correctness of these features, i.e., the ability of each model to infer genuine associations and disentangle them from the spurious ones.

**Figure 3:**
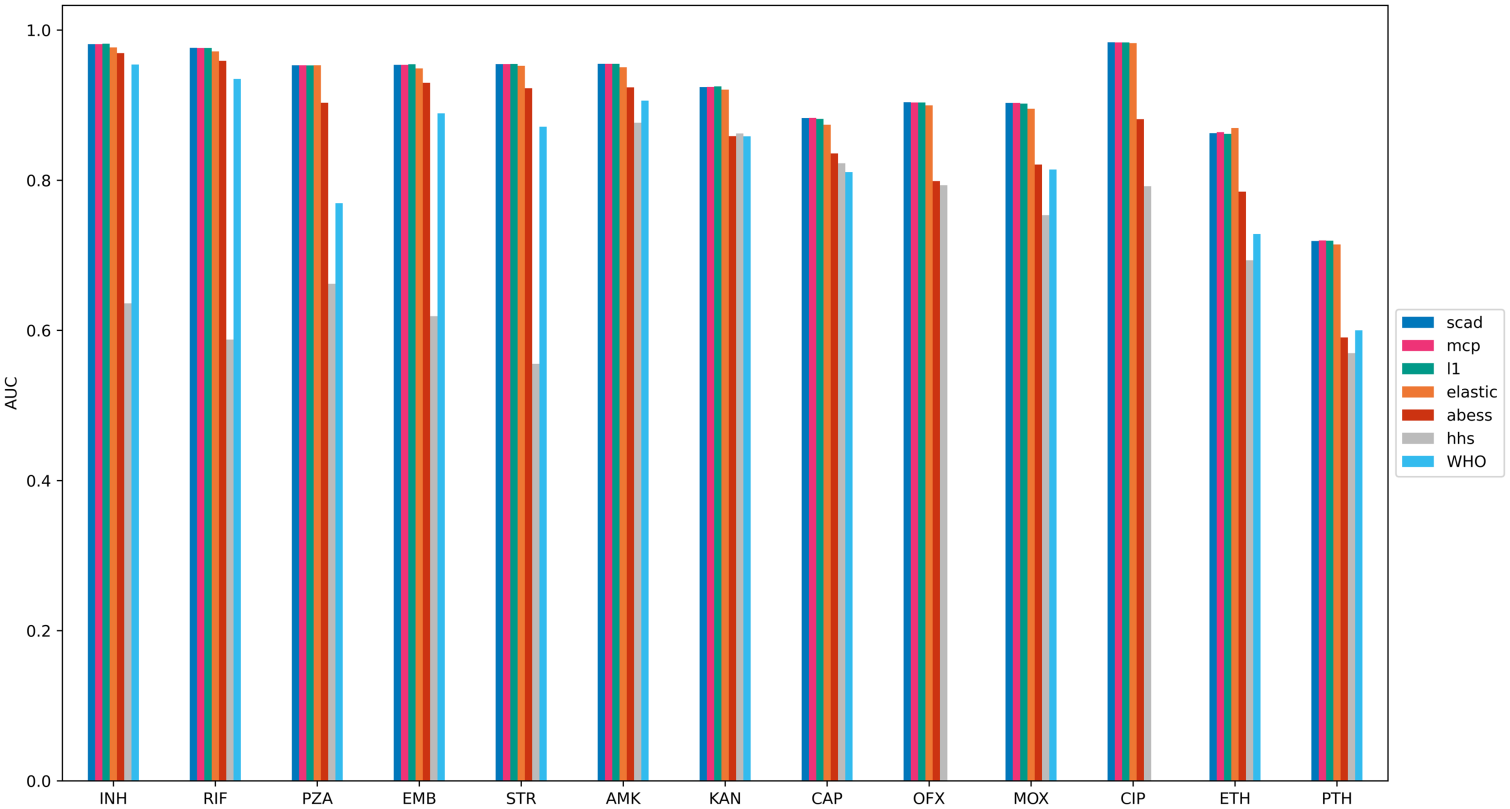
Drug resistance prediction accuracies of compared ML methods. Logistic regression models with SCAD, MCP, elastic net and L1 regularization outperform other methods. The figure shows the average ROC AUC scores of logistic regression models trained on features majorly selected by regression with L1 (teal), MCP (magenda) and SCAD (blue) regularization, elastic net (orange), HHS (grey), ABESS (red) and WHO catalog (cyan). Feature selection models were trained on the training parts of 5 dataset splits, and features selected in at least three of the splits were included. Logistic regression was trained using these selected features on the training set, and ROC AUC was estimated on the testing set. The ROC AUC scores were averaged over all dataset splits.

To assess the prediction quality of the selected feature sets, logistic regression models were trained on the training sets using the features chosen by each method, and prediction performance was evaluated using ROC AUC on the testing sets. For comparison, we also evaluated the prediction quality of the DA method based on the WHO catalog (Walker et al., 2022). Feature sets obtained by the logistic regression with L1, SCAD, MCP and elastic net regularization techniques exhibited similar prediction quality, whereas HHS performed the worst, except for capreomycin and amikacin. The DA method based on WHO catalog showed lower prediction quality for all drugs compared to all methods, except for HHS, which yielded the worst results for most drugs (Figure 3 and Supplementary Tables S18–S24). This outcome was expected since the catalog was constructed by applying stringent statistical thresholds to label mutations as “associated with resistance,” resulting in higher specificity and lower sensitivity (Supplementary Tables S18–S24), and being overly conservative in its predictions.

We also compared the number of selected features and the stability of selection by different methods (Supplementary Table S25). The number of selected features varied among the algorithms with logistic regression with different regularization techniques typically picking more features than ABESS and HHS. When comparing the number of features selected in at least one dataset split with the number of majorly selected features (selected in at least three splits), surprisingly, elastic net demonstrated the largest fraction of features selected in 3 and more splits, demonstrating relatively high stability. However, these sets were still too large to interpret, while the second leader - ABESS - majorly selected the small number of features (Supplementary Table S25).

Next, we examined the correctness of selected features. Assuming that most of each drug’s phenotype could be explained by mutations in a relatively small number of genes involved in anti-TB drug action, we compared the methods based on their ability to correctly identify known associations of drugs with genes. Below, we refer to a gene as a “gene associated with a given drug” if this gene was associated with resistance to given drug in previous studies (Supplementary Table S1). We refer to a gene as a “gene selected by the method for a given drug” if at least one mutation in this gene was majorly selected by this method for this drug. We compared methods by their ability to select features corresponding to genes known to be associated with resistance to a particular drug while avoiding features associated with other drugs. For this, we considered only genes whose mutations are known to be associated with resistance to some drugs, i.e., present in Supplementary Table S1. Thus, for each drug, each gene can be selected or not selected by the tested method, and, on the other hand, be associated or not associated with resistance to this drug in Supplementary Table S1. Therefore, a gene is a “true positive” (TP) if it was selected by the method for a given drug and is associated with this drug, a “false positive”(FP) if it was selected but is not associated, a “false negative”(FN) if it was not selected but is associated, and a “true negative”(TN) if it was not selected and is not associated with this drug. If a gene is associated with multiple drugs, it is considered either TP or FN for each drug depending on whether it was selected by the algorithm.

The first comparison criterion is PPV (Table 2). In our case, it is the number of genes associated with a given drug selected by the method (TP) divided by the number of all genes selected for the particular drug (TP + FP) among genes associated with resistance to any of the drugs. ABESS exhibited the highest PPV for 8 antibiotics, while HHS was the best for five drugs, logistic regression with elastic net regularization performed best for prothionamide. Logistic regression with L1, SCAD and MCP regularization showed the lowest PPVs.

**Table 2:** Comparison of the ability of feature selection methods to correctly identify genes involved in drug resistance.

Metric	PPV						Sensitivity						Specificity					
Method	L1	SCAD	MCP	Elastic	ABESS	HHS	L1	SCAD	MCP	Elastic	ABESS	HHS	L1	SCAD	MCP	Elastic	ABESS	HHS
Rifampicin	0.09	0.09	0.09	0.11	**0.50**	0.33	**0.50**	**0.50**	**0.50**	**0.50**	**0.50**	**0.50**	0.68	0.68	0.68	0.74	**0.97**	0.94
Isoniazid	0.38	0.40	0.40	0.33	0.75	**1.00**	**1.00**	0.80	0.80	0.60	0.60	0.20	0.71	0.79	0.79	0.79	0.96	**1.00**
Pyrazinamide	0.11	0.11	0.11	0.09	**0.50**	0.08	**0.33**	**0.33**	**0.33**	**0.33**	**0.33**	**0.33**	0.73	0.73	0.73	0.67	**0.97**	0.63
Ethambutol	0.25	0.25	0.25	0.10	**0.50**	0.33	**0.33**	**0.33**	**0.33**	0.11	0.11	0.11	0.63	0.63	0.63	0.63	**0.96**	0.92
Streptomycin	0.33	0.33	0.33	0.30	**0.50**	0.33	**1.00**	**1.00**	**1.00**	**1.00**	0.67	0.67	0.80	0.80	0.80	0.77	**0.93**	0.87
Kanamycin	0.17	0.13	0.13	0.15	0.67	**1.00**	**1.00**	**1.00**	**1.00**	**1.00**	**1.00**	**1.00**	0.68	0.58	0.58	0.65	0.97	**1.00**
Amikacin	0.17	0.17	0.17	0.11	**1.00**	0.50	**1.00**	**1.00**	**1.00**	0.50	0.50	0.50	0.68	0.68	0.68	0.74	**1.00**	0.97
Capreomycin	0.15	0.13	0.13	0.13	0.50	**1.00**	**1.00**	**1.00**	**1.00**	0.50	0.50	1.00	0.65	0.58	0.58	0.77	0.97	**1.00**
Ofloxacin	0.13	0.13	0.11	0.14	**1.00**	0.33	**1.00**	**1.00**	**1.00**	**1.00**	0.50	0.50	0.58	0.58	0.48	0.61	**1.00**	0.94
Moxifloxacin	0.13	0.13	0.13	0.09	0.50	**1.00**	**1.00**	**1.00**	**1.00**	0.50	0.50	0.50	0.58	0.55	0.55	0.68	0.97	**1.00**
Ciprofloxacin	0.11	0.11	0.11	0.13	**1.00**	**1.00**	**0.50**	**0.50**	**0.50**	**0.50**	**0.50**	**0.50**	0.74	0.74	0.74	0.77	**1.00**	**1.00**
Ethionamide	0.18	0.20	0.20	0.20	**0.67**	0.00	**0.67**	**0.67**	**0.67**	**0.67**	**0.67**	0.00	0.70	0.73	0.73	0.73	**0.97**	**0.97**
Prothionamide	0.11	0.13	0.12	**0.13**	0.00	0.00	**0.67**	**0.67**	**0.67**	**0.67**	0.00	0.00	0.47	0.57	0.50	0.53	**0.97**	**0.97**

The methods that incorporate a parsimony assumption, namely ABESS and HHS, demonstrate better PPV and Specificity statistics, indicating that they are less likely to select genes known to be associated with other drugs compared with regularized logistic regression methods. Below, we refer to a gene as a “gene associated with a given drug,” if it has been associated with resistance to that drug in previous studies (Supplementary Table S1). We refer to a gene as a “gene selected by the method for given drug” if at least one mutation in this gene was majorly selected by the method for that drug. Only genes associated with at least one drug, i.e., those present in Supplementary Table S1, are considered. Thus, for each drug, each gene can either be selected or not selected by the method, and can also be associated or not associated with the drug. A gene is a “true positive” if it was selected by the method for a given drug and is associated with that drug, a “false positive” if it was selected but not associated with the drug, a “false negative” if it was not selected but is associated, and a “true negative” if it was not selected and is not associated with the drug. If a gene is associated with multiple drugs, it is considered either TP or FN for each of the drugs depending on whether it was selected by the algorithm. Maximum values for all drugs and all metrics are highlighted in bold.

We also examined two additional metrics to emphasize the strength and weakness of each method: sensitivity (Sn = TP/(TP + FN)) and specificity (Sp = TN/(TN + FP)). The values of sensitivities were the same or better for logistic regression methods compared to ABESS and HHS. However, the specificity values were consistently lower for these methods (Table 2).

The genes selected by ABESS generally are in agreement with the literature (Table 3 and Supplementary Table S1). It is also noteworthy that ABESS predominantly selects PFAM domain features, constituting approximately 53% of all majorly selected features (Table 3).

**Table 3:** Mutations (relative to H37Rv) and aggregated features which are majorly selected by ABESS.

Drug name	Gene	Position	Type of event	Reference allele	Alternative allele	Number of splits in which the feature was selected
Rifampicin	*eccC2*	258	SNV	R	P	5
	***rpoB***	435	SNV	D	V	5
	***rpoB***	450	SNV	S	L	5
	***rpoB***	–	PF04565 is changed	–	–	5
	*fadA*	–	PF00108 is changed	–	–	4
	*katG*	–	PF00141 is changed	–	–	4
	***rpoB***	435	SNV	D	Y	3
Isoniazid	***katG***	–	PF00141 is changed	–	–	5
	***katG***	315	SNV	S	T	5
	***fabG1***	−15	SNV	C	T	5
	*rpoB*	–	PF04565 is changed	–	–	4
	***inhA***	–	PF13561 is changed	–	–	4
	*pepC*	–	PF02127 is changed	–	–	3
Pyrazinamide	***pncA***	–	PF00857 is changed	–	–	5
	*fadA*	–	PF00108 is changed	–	–	5
	*Rv2017*	262	SNV	A	E	4
	*Rv2017*	–	PF06114 is changed	–	–	4
	*ctpB*	–	PF00403 is changed	–	–	4
	*Rv0108c*	−73	Insertion	T	GT	4
	*nadD*	−44	SNV	T	G	4
	*rpoB*	–	PF04565 is changed	–	–	4
	*Rv2585c*	462	SNV	C	S	3
Ethambutol	***embB***	406	SNV	G	A	5
	***embB***	–	PF04602 is changed	–	–	5
	*rpoB*	–	PF04565 is changed	–	–	5
	***embB***	406	SNV	G	S	4
	*ctpB*	–	PF00403 is changed	–	–	3
	*eccC2*	258	SNV	R	P	3
	*fadA*	–	PF00108 is changed	–	–	3
Streptomycin	*inhA*	–	PF13561 is changed	–	–	5
	*katG*	315	SNV	S	T	5
	*katG*	–	PF00141 is changed	–	–	5
	***rpsL***	–	PF00164 is changed	–	–	5
	***rrs***	514	SNV	A	C	5
Kanamycin	*gyrA*	–	PF00521 is changed	–	–	5
	***rrs***	1,401	SNV	A	G	5
	***eis***	−10	SNV	C	T	3
	***eis***	−14	SNV	G	A	3
	*htrA*	−33	Insertion	T	GT	3
Amikacin	***rrs***	1,401	SNV	A	G	5
Capreomycin	***rrs***	1,401	SNV	A	G	5
	*fabG1*	−15	SNV	C	T	4
Ofloxacin	***gyrA***	–	PF00521 is changed	–	–	5
	*Rv1897c*	–	PF02580 is changed	–	–	3
	***gyrA***	91	SNV	S	P	3
Moxifloxacin	***gyrA***	–	PF00521 is changed	–	–	5
	*katG*	–	PF00141 is changed	–	–	4
	***gyrA***	91	SNV	S	P	3
Ciprofloxacin	*pks8*	1,357	SNV	A	T	5
	***gyrA***	–	PF00521 is changed	–	–	3
Ethionamide	*fabG1*	−15	SNV	C	T	4
	***inhA***	–	PF13561 is changed	–	–	4
	***ethA***	–	PF00743 is changed	–	–	3
	*fbpC*	–	PF00756 is changed	–	–	3
Prothionamide	*pncA*	–	PF00857 is changed	–	–	3

Genes associated with resistance to the given drug in previous studies (Supplementary Table S1) and correctly assigned to this drug by ABESS are marked in bold, improperly assigned genes are underscored.

### ABESS does not select features clearly associated with the population structure

Structural features were generated for all drugs by TreeBraker (see “Methods,” Supplementary Table S26). The number of structural features varied from 1 for prothionamide to 163 for rifampicin (see Supplementary Table S27). To assess whether the phylogenetic features correspond to specific geographic locations or MTB phylogenetic lineages, we assigned geographic locations to 3,541 isolates (about 29% of the dataset) for which this information was available, and lineage information to all but two isolates, for which lineage-defining mutations from the TB Profiler table were found (Supplementary Table S28). Across all drugs, for 139 (about 21%) of all structural features, the distributions of isolates having these features by location categories were significantly different from the distributions by location categories of all isolates with known phenotypic states for corresponding drugs. For 480 (about 69%) of all structural features, the distributions of isolates by MTB phylogenetic lineages were significantly different from those of all isolates with known phenotypic states for corresponding drugs (see Supplementary Tables S28, S29). These findings indicate strong geographic and phylogenetic signals in the structural features and, consequently, in the dataset.

All structural features were added to the set of genetic features for each corresponding drug, and the compound feature sets were used for feature selection with the ABESS method. None of the structural features were majorly selected (Supplementary Table S30), and none of the selected SNVs were from the TB-Profiler catalog of lineage-defining SNVs used for genotyping (Napier et al., 2020). Therefore, the population structure of the data did not appear to compromise the set of features selected by ABESS.

### New associations can be found by repeating the search on the unexplained resistant isolates

Features majorly selected by ABESS did not fully explain resistance for several drugs. A significant percentage of isolates, especially for second-line drugs, had resistant phenotypes that were not explained by mutations selected in three or more folds (Table 4). To address this, we performed a second iteration of ABESS on a reduced dataset containing resistant isolates which were not explained by majorly selected features in the first iteration (Supplementary Table S31) (see “Methods”). For six drugs, the second iteration increased the number of isolates with correctly explained resistant phenotype, with increases ranging from 0.38 to 24.26% (Table 4). Among the 26 features majorly selected at the second iteration, 19 were known from the literature as DR-associated mutations or domains in the DR-associated genes, 16 of these were correctly predicted for the corresponding drugs (Table 5). Two drug-associated genes that were missed in the first iteration (*embA* for ethambutol and *gid* for streptomycin) were identified in the second iteration.

**Table 4:** The second iteration of ABESS improves sensitivity of DR predictions.

Drug name	Number of resistant isolates	Number of isolates participated in the first ABESS iteration	Fraction of resistant isolates explained by the first ABESS iteration	Number of resistant isolates unexplained after the first ABESS iteration	Number of isolates participated in the second ABESS iteration	Fraction of resistant isolates explained by the first and the second ABESS iteration	Number of resistant isolates unexplained after the second ABESS iteration
Rifampicin	2,277	9,319	**89.92%**	230	939	**91.71%**	189
Isoniazid	2,754	9,252	**90.39%**	265	888	**90.77%**	254
Pyrazinamide	884	7,334	**55.50%**	393	3,263	**67.78%**	285
Ethambutol	1,223	8,143	**74.74%**	309	2,057	**86.71%**	163
Streptomycin	1,344	3,769	**86.12%**	187	523	**93.79%**	83
Kanamycin	275	961	79.65%	56	195	79.65%	56
Amikacin	322	1,482	71.96%	90	415	71.96%	90
Capreomycin	323	1,547	**63.37%**	118	566	**65.59%**	111
Ofloxacin	376	1,559	69.36%	115	477	69.36%	115
Moxifloxacin	206	964	61.96%	78	366	61.96%	78
Ciprofloxacin	81	366	**54.70%**	37	165	**78.96%**	17
Ethionamide	222	553	67.96%	71	177	67.96%	71
Prothionamide	144	351	**28.89%**	102	249	**43.61%**	81

We defined a resistant isolate as being explained by ABESS on a given dataset split if logistiс regression trained on the training set of this split using the features majorly selected by ABESS, classifies this isolate as resistant. All the numbers were averaged across splits. If the fraction of explained isolates for a given drug increases, the values after the first and the second iterations are marked in bold.

**Table 5:** Additional mutations (relative to H37Rv) majorly selected by ABESS on the second iteration.

Drug name	Gene	Position	Type of event	Reference allele	Alternative allele	Number of splits in which the feature was selected
Rifampicin	*eccC2*	258	SNV	R	P	4
***Rifampicin***	***rpoB***	170	SNV	V	F	3
***Rifampicin***	***rpoB***	430	SNV	L	P	3
Isoniazid	***fabG1***	−17	SNV	G	T	4
Pyrazinamide	*embB*	306	SNV	M	I	5
Pyrazinamide	*fadA*	–	PF00108 is changed	–	–	5
***Pyrazinamide***	***pncA***	–	Broken	–	–	5
***Pyrazinamide***	***pncA***	−11	SNV	T	C	5
*Pyrazinamide*	*embB*	306	SNV	M	V	4
Pyrazinamide	*Rv0658c*	75	SNV	L	P	3
Pyrazinamide	*Rv2585c*	462	SNV	C	S	3
Ethambutol	***embB***	306	SNV	M	I	5
***Ethambutol***	***embB***	306	SNV	M	V	5
***Ethambutol***	***embA***	−12	SNV	C	T	4
***Ethambutol***	***embB***	1,002	SNV	H	R	4
***Ethambutol***	***embB***	1,024	SNV	D	N	3
Ethambutol	*Rv0012*	233	SNV	C	R	3
Streptomycin	***gid***	–	PF02527 is changed	–	–	5
***Streptomycin***	***gid***	92	SNV	E	D	3
Kanamycin	*gyrA*	–	PF00521 is changed	–	–	3
Capreomycin	***rrs***	1,484	SNV	G	T	3
Moxifloxacin	*vapB1*	−2	Insertion	C	AGCGCTGTTCTGGCGCTAATCTGACGCTAGAATAGCGC	3
Ciprofloxacin	***gyrA***	–	PF00521 is changed	–	–	4
***Ciprofloxacin***	***gyrA***	94	SNV	D	G	3
Ethionamide	*Rv0221*	–	PF06974 is changed	–	–	3
Prothionamide	***ethA***	–	PF13450 is changed	–	–	3

The method identified many rare mutations properly associated with the corresponding drugs, which were missed in the first iteration. Pairs of drugs and genes that were associated with each other in previous studies (Table 1) are marked in bold, while pairs in which the gene was associated with a different drug are underscored.

We attempted to apply this approach to the other methods tested above. All variants of logistic regression turned out to majorly select so many features, that these features explained all resistant isolates in the first iteration. This came at the cost of mixing correct genes with the ones associated with other drugs, according to the literature (Table 2). Meanwhile, HHS, which performed the worst in terms of ROC AUC (Figure 1), explained much fewer resistant isolates on average compared to ABESS. Although the percentage of explained resistant isolates increased significantly after the second iteration for all drugs, these values remained lower than those for ABESS for all drugs except pyrazinamide, kanamycin, amikacin, capreomycin and ethionamide (Table 4 and Supplementary Table S33). The second iteration enabled HHS to select relevant mutations: of the 43 features majorly selected in the second iteration, 19 were point mutations and 19 were change of domain events, 4 of which were in DR-associated genes known from the literature (Tables S33-S34). Twenty of these 43 features were in genes selected in the first iteration, including one domain change event; 15 domain events were selected in new genes. Three of the domain features selected in the second iteration were in known drug associated genes not selected in the first iteration: the PF01751 PFAM domain change event in the *gyrB* gene was selected for ofloxacin, the PF00743 PFAM domain change event in the *ethA* was selected for ethionamide and the PF00141 PFAM domain change event in *katG* was selected for isoniazid. The rpsL K88R was selected for streptomycin. Thus, the second iteration allowed HHS to identify *gyrB, katG, rpsL and ethA*, which had not been selected in the first iteration. However, compared to ABESS, HHS tended to select more genes that were not associated with any drug in the literature.

## Discussion

Often, the same ML methods are applied to address two related yet distinct tasks: solving classification problems, such as predicting drug genotypic states based on genotypes in our case, and performing feature selection, such as identifying mutations reliably associated with drug resistance. However, a method that is optimal for one task may be unsuitable for another. The primary aim of our study was to identify the most effective ML method for uncovering new genes and mutations associated with resistance to anti-TB drugs. We evaluated various methods for analyzing the relationships between mutations in clinical isolates and their corresponding phenotypic states across different drugs. The task was particularly challenging due to the high correlation among phenotypic states for different drugs, which can obscure true weak associations between features and proper drug phenotypes. However, for classification tasks, such correlations are less problematic as long as the method is applied to datasets with similar statistical properties to the training set.

Among the tested methods, ABESS proves to be the most suitable for addressing the challenge of correlating response variables, as it effectively identifies genuine, experimentally confirmed associations between drugs and genes. However, all compared regularized logistic regression methods outperform ABESS in terms of a phenotype prediction accuracy. Nevertheless, ABESS achieves higher classification accuracy than the HHS method (Libiseller-Egger et al., 2020) or the direct association method based on the 2021 WHO mutation catalog, which is overly conservative (Walker et al., 2022). While ABESS is the most accurate for uncovering proper functional associations between mutations and drugs, it yields the fewest novel genes potentially involved in drug resistance. In total, 16 novel genes have been selected as markers of drug resistance or susceptibility: 12 in the first iteration, and an additional 4 in the second iteration of ABESS.

Five features majorly selected by ABESS corresponded to four genes: *pepC*, *Rv2017*, *fadA* and *vapB1*, each exhibiting positive regression coefficients. These features were predictive of resistant phenotypes to INH, PZA and MOX (Tables 4, 5 and Supplementary Tables S12, S31). We can infer potential mechanisms through which these genes contribute to MTB’s adaptation to drug actions. Protease *pepC* is shown to be expressed during pellicle growth and is involved in virulence (Kerns et al., 2014). *Rv2017* was annotated as a potential transcriptional regulatory protein, it was also identified as the antitoxin component of the dual toxin/antitoxin pair *Rv2016*/*Rv2017* (Akarsu et al., 2019). *Rv2017* was identified as an essential gene in certain MTB strains but not in others (Bosch et al., 2021). Notably, two features, SNV and a domain change in *Rv2017*, were selected as predictors of resistance to pyrazinamide. *VapB1,* an antitoxin belonging to the VapBC family, undergoes degradation under unfavorable conditions, leading to toxin *VapC1* release and subsequent inhibition of cell growth (Lu et al., 2016). An insertion upstream of *vapB1,* potentially affecting gene expression, was selected as moxifloxacin resistance marker. Our findings are in agreement with previously suggested important roles of toxin/antitoxin systems in MTB’s adaptation to drug-induced stress through a transition to the non-replicative persistent state (Khan et al., 2023). Small peptides that mimic the structures of interfaces of cognate toxin-antitoxin pairs, disrupting their complexes formation by promoting toxin ribonuclease activity, could be explored as potential new anti-TB drugs (Srivastava et al., 2021). *fadA* encodes a possible acyl-CoA thiolase and is involved in lipid degradation. In our study, a change in *fadA* N-terminal domain was identified as a resistance marker for pyrazinamide. *fadA* was also associated with ofloxacin and kanamycin resistance in one of the early GWAS studies on MTB (Zhang et al., 2013). Additionally, *fadA* expression is induced in log-phase MTB upon exposure to isoniazid (Abo-Kadoum et al., 2021). Moreover, *fadA* is secreted under hypoxic conditions and is enriched in granulomas, where it functions as an acetyltransferase, converting host acetyl-CoA to acetoacetyl-CoA. The reduction in acetyl-CoA level promotes granuloma progression (Yang et al., 2021). *FadA,* along with other enzymes involved in fatty acid *β*-oxidation, is strongly upregulated under starvation stress in non-replicative persisters due to a metabolic shift from glycolysis to lipid catabolism and ketone body metabolism. Starved persisters are more viable in acidic growth conditions and under antibiotic stress (Davis et al., 2024). Notably, pyrazinamide targets CoA biosynthesis and remains the only drug active against non-replicative persisters (Lamont and Baughn, 2019). Targeting MTB lipid metabolism is now considered a promising approach for adjunctive therapy (Davis et al., 2024; Kim and Shin, 2023). Overall, novel genes selected by ABESS are likely to be compensatory and, in fact, associated with survival in the host and drug tolerance.

The HHS method identified 107 novel genes after the first iteration and 21 genes after the second iteration. All features selected by HHS exhibited positive regression coefficients and, therefore, were predictive of resistant phenotypic states (tables S13, S34). After additional filtering of selected features by Fisher’s *p*-value (p-value < 0.05) 44 and 1 genes remained for the first and second iteration, correspondingly. 24 of these genes were identified by HHS as associated with resistance to pyrazinamide. There was no overlap in the selected sets of novel genes between HHS and ABESS. However, the HHS output also yielded some interesting findings.

For instance, the HHS method recovers *rpoC* association with streptomycin and rifampicin resistance. Interestingly, associated variants are not the same for these drugs: a single-residue replacement at site 402 of *rpoC* was selected as rifampicin resistance marker, while streptomycin resistance was associated with a replacement at site 516 of the same gene. Mutations in several sites in *rpoC* were previously shown to have a compensatory role for rifampicin resistance (Comas et al., 2012) and Q1126K replacement in *rpoC* was beneficial in rifampicin and streptomycin double-resistant but not in sensitive strains (Moura et al., 2017). HHS predicted an association between *rpoA*, another RNA polymerase subunit, variant and pyrazinamide resistance. The selected variant, V183G, is known to be compensatory for mutations in *rpoB* conferring rifampicin resistance (Comas et al., 2012). It has been shown recently that MDR strains with RNAP compensatory mutations carry more drug resistance mutations and could be transmitted at least as effectively as wild-type strains (Goig et al., 2023; Loiseau et al., 2023; Merker et al., 2018).

The set of novel genes predicted for pyrazinamide resistance also includes 4 membrane transport proteins: *cycA* (involved in D-alanine, D-serine and glycine transport), *dppC* (probable ABC transporter of dipeptide across the membrane), *sugI* (involved in sugar transport) and *cysT* (probable sulfate-transport ABC transporter) (Kapopoulou et al., 2011). *cycA* and *sugI* variants were previously associated with D-cycloserine resistance, the drug which is commonly used to treat MDR and XDR-TB (Chen et al., 2012; Chen et al., 2017).

Additionally, *nusG* variant is predicted to be associated with rifampicin resistance. *NusG* is a probable transcription antitermination protein which interacts with rho factor and RNA polymerase. Thus, the selected variant is likely to be compensatory for *rpoB* mutations and *nusG* regulatory mutation was previously shown to be compensatory in rifampicin and streptomycin double-resistant genotypes (Moura et al., 2017).

*rplU*, which encodes 50S ribosomal protein L21, is predicted by HHS to be associated with streptomycin resistance. Notably, another ribosomal protein, RpsL, and ribosomal RNA *rrs* are known targets of streptomycin (Table 1). *pntAa* is predicted by HHS to be associated with streptomycin resistance and its annotated function as a proton pump suggests its possible role in MDR acquisition.

Logistic regression methods selected the largest set of novel genes, with over 500 novel genes remaining after raw p-value filtering for each regularization technique. These large sets lack biological interpretation proving this group of methods may not be suitable for GWAS, despite the fact that these methods provide the highest prediction quality (Figure 3).

Even the most effective algorithm among those studied, ABESS, was unable to fully disentangle the cross-associations of features to different drugs (Table 3). For example, *rpoB* was selected for isoniazid, ethambutol and pyrazinamide while *katG* was selected for rifampicin. Also, when an isolate is resistant to a certain second-line drug, it is typically resistant to one or more first-line drugs. Again, such co-resistance leads to cross-associations between genes and both first and second-line drugs: *katG* was selected for streptomycin and moxifloxacin, *pncA* was selected for prothionamide, *fabG1* was selected for capreomycin and ethionamide. Cross-associations between second-line drug features were also observed, such as the change of *gyrA* domain being selected for kanamycin. Recently new deep learning ML methods were suggested for predicting MTB drug resistance, e.g., hierarchical recurrent neural networks with attention, transformers and convolutional neural networks (Kuang et al., 2022; Jiang et al., 2022). Generally, these methods have better accuracy of DR prediction compared to classical ML methods for large datasets, however these methods as well suffer from correlations of features and predict spurious associations of mutations and drugs if they were applied for the goal of feature selection (Kuang et al., 2022; Jiang et al., 2022).

The prediction quality of ML algorithms is limited by the quality of the datasets. One contributing factor is that patients may be infected by multiple strains, which leads to errors in both genotyping and phenotyping. Further, assigning discrete phenotypes becomes challenging in cases of intermediate drug resistance, where resistant isolates with low growth rates may be misinterpreted as susceptible (Bradley et al., 2015). There is growing evidence that MICs are more informative for GWAS than binary phenotypic labels (Ahmad et al., 2016). Differences in culturing and measurement protocols further contribute to inconsistencies in phenotyping, undermining the reproducibility of results. For some drugs, phenotypic tests are now considered less reliable than sequencing of known drug-associated genes, for example, ethambutol, pyrazinamide, ethionamide and prothionamide (World Health Organization, 2021). Epistasis may change the phenotypic effects of mutations associated with resistance. For instance, *eis* promoter mutations do not confer amikacin or kanamycin resistance if co-occurred with loss-of-function mutations in the *eis* coding region (Organization WH, 2023). Our model does not account for epistatic interactions between mutations and it is unable to correctly classify cases of sign epistasis. These limitations significantly hinder the ability to assess the completeness of our understanding of genetics underlying drug resistance. The CRyPTIC study, which used a high-quality phenotype dataset, demonstrated that an additive model of genetic effects explains MIC variance better than binary phenotype variance, albeit still imperfectly for most drugs (Consortium TCr, 2022). This study also proposed new resistance mechanisms, including one involving the toxin-antitoxin VapBC20 system, which is in line with our results.

Overall, our study demonstrates the effectiveness of advanced feature selection alongside feature aggregation with subsequent filtering by frequency. Notably, aggregated features were majorly selected and comprised a significant proportion of all selected features. Another advantage of the aggregation is the possibility to take into account the effects of rare mutations without directly including them into the model, which increases both the robustness and training speed. Moreover, advanced feature selection is instrumental in addressing the problem of correlations between phenotypes. Unlike simpler methods, which tend to attribute resistance to one drug to mutations in genes associated with resistance to others, the proposed strategy offers a more nuanced approach. Importantly, our selection strategy mitigates biases associated with population structure, as well. Despite the presence of a strong geographic signal in our data, the best algorithm did not select any population structure-associated features.

## Data Availability

The dataset unifies characterized whole-genome sequences of *M. tuberculosis* from multiple studies (Walker et al., 2015; Farhat et al., 2019; Zhang et al., 2013; Farhat et al., 2013; Coll et al., 2018; Yang et al., 2017; Casali et al., 2014; Johnsen et al., 2019; Kouchaki et al., 2019; Pankhurst et al., 2016). Short Illumina reads are available in public repositories (SRA or ENA). Sample ids, phenotypes and links to the source papers are summarized and listed in Supplementary Table S2. The rest of the dataset and the source code can be downloaded from the GitHub repository: https://github.com/Reshetnikoff/m.tuberculosis-research-code.

## Footnotes

^1^ https://www.ebi.ac.uk/interpro/
