# Supplementary material for: Feature selection and aggregation for antibiotic resistance GWAS in Mycobacterium tuberculosis: a comparative study
Source: Front Microbiol. 2025 Jun 18;16:1586476. doi: 10.3389/fmicb.2025.1586476 (PMC12213493; doi:10.3389/fmicb.2025.1586476)
Supplement: Supplementary file 1 [file Data_Sheet_1.pdf]

Supplementary Materials.

Supplementary Methods

Dataset and raw data processing

The dataset contains 13258 whole-genome sequences of *M. tuberculosis* for which both phenotypes and raw data (Illumina short reads) were available from the literature [1–10]. TruSeq3-PE-2 adapters and low-quality ends (Q<30 with sliding window of length 10) were trimmed using trimmomatic v.0.38 (java -Xmx1800m -jar trimmomatic-0.38.jar PE -phred33 R1.fastq.gz R2.fastq.gz out_p1.fastq.gz out_u1.fastq.gz out_p2.fastq.gz out_u2.fastq.gz ILLUMINACLIP:TruSeq3-PE-2.fa:1:30:10 SLIDINGWINDOW:10:30). Next, raw reads were mapped onto the reference genome H37Rv (version 3) by bwa mem with default parameters and resulting bam files were sorted and indexed with samtools. Then we marked duplicates using MarkDuplicates for the Picard package. In cases when multiple runs corresponded to one biosample we merged such bam files in one after the previous step.

Variant calling was performed with HaplotypeCaller from the GATK suite (version 4) [11]. Variants were called jointly using GenomicsDBImport and GenotypeGVCFs tools: at first, gvcf files were produced with HaplotypeCaller, then multiple gvcfs were imported to GenomicsDB and, finally, the db was jointly genotyped with GenotypeGVCFs producing joint vcf. Since joint calling of the whole dataset was computationally infeasible, we've split the dataset to three subsets, genotyped all the subsets jointly and then split joint vcfs to individual ones. After that, multiple filtering stages were performed.

At first, the variants were filtered by quality with recommended parameters (hard filtering with QD >= 2, FS <=60, MQ >= 40, MQRankSum >= -12.5, ReadPosRankSum >= -8, SOR <= 3). After that only variants covered with at least 10 reads (DP >= 10) and having allele frequency higher than 75% were keeped. Variants from regions annotated as "Repeat_region" or "mobile_element" according to Mycobrowser v.3 annotation were also removed.

At the second stage both variants and isolates were filtered with iterative procedure: we first delete all variants, which are covered with less than 10 reads in less than 90% of the isolates, from all the isolates; then we delete all isolates in which less than 90% of variable loci (preserved on previous iteration) are covered with at least 10 reads. This was repeated until convergence. This left us with 12354 sequences for further analysis.

Next step was the annotation based on Mycobrowser v.3. To take into account possible start codon loss or appearance of the inframe stop codons along with frameshifts and correctly annotate SNVs, insertions and deletions, we've performed a special translation procedure. For each isolate we took the nucleotide sequence of each protein-coding gene along with its

180 bp upstream and downstream regions which differed from the reference sequence and ran blastx [12] to compare this nucleotide sequence with the reference protein sequence of a given gene. Next, the first start codon upstream of the first position of the best blastx hit among all reading frames was found. The isolate's nucleotide sequence was translated from this start codon to the first stop codon within the same reading frame. The protein sequence obtained was aligned to the reference protein sequence with Needleman–Wunsch algorithm implemented in biopython [13]. The gene was labeled as "broken" if it was shorter than half the reference protein length or 30% longer than the reference protein. If the gene is broken, no mutation is considered in this gene. Otherwise, all mutations inside the gene are annotated according to position in protein based on the alignment. SNVs, insertions and deletions at a distance less than 100 bp upstream of a gene were annotated as upstream relative to this gene. Nucleotide variants in noncoding genes were annotated using the reference genome gene boundaries. Synonymous mutations were excluded from the further analysis.

Splitting dataset for further experiments

The final dataset was split to 13 smaller datasets containing only isolates with known phenotypes for each antibiotic. Each isolate is typically a part of multiple datasets. All feature aggregation, parameter tuning, feature selection and quality estimation procedures were based on 5-fold cross-validation for each drug separately. For each drug the dataset was randomly split into four non overlapping subsets (folds). Each fold was used as the testing set one at a time, while the union of the others served as the training set. Below we call every such split "the dataset split". For every dataset split the testing fold is called "the testing set" and the union of the others is called "the training set".

Aggregation of mutations

We examined three types of aggregated features: an indicator of the presence of any mutation in a gene, an indicator that the gene is broken, i.e. it has a loss of function mutation, and features indicating alternation of PFAM domains.

If there is any mutation in a gene then the indicator of the presence of mutations in this gene takes the value of "1" and "0" otherwise. We call this approach "gene aggregation". "Broken gene" is another binary feature. Gene is considered to be 'broken' if the length of its open reading frame (ORF) is decreased due to mutations by half or increased by 30% relative to the annotated ORF length (see Dataset and raw data processing). The design of "the domain X has been changed" features is described in more detail in the next section.

Then the aggregated features generated mutations and the other binary features that had happened less than 3 times were excluded from the dataset. Then, the features whose values fully coincide, i.e. having correlation equal to 1, on the training set of the given dataset split were unified and were treated as one feature during the selection process. For

presentation purposes the same numerical ID was assigned to all features constituting unified ones.

PFAM domains based feature aggregation

Gene names from the Mycobrowser v.3 were used to get domains' markup and pretrained HMM models from PFAM database (PFAM-A 33.1 version). For each isolate, HMM scores were computed for all mutated sequences of domains by using the maximum a posteriori method. The python module “pomegranate” (v. 0.12.2) was used to implement a scoring function for trained HMM models: the score was a logarithm of probability that the trained model generated a given sequence.

To move from continuous HMM scores to binary features for each drug and each domain and each dataset split we found a threshold for HMM score. The isolates from the training set were ordered by the Δscore - an absolute difference between an HMM score of the domain of an isolate and a corresponding HMM score of the H37Rv domain sequence. The threshold was selected to split the dataset maximizing the difference of fractions of susceptible and resistant isolates:

$$threshold = argmax_{threshold} abs(f_S(\Delta score \geq threshold) - f_R(\Delta score \geq threshold))$$

where, $f_S(\Delta score \geq threshold)$ is a fraction of susceptible isolates with a Δscore greater or equal to the threshold, $f_R(\Delta score \geq threshold)$ is an analogously defined fraction of resistant isolates.

Accordingly, for each isolate and each drug and each dataset split we labeled a domain with HMM score deviating from the wild-type score more or equal to the threshold found as “changed” so that an additional 4721 binary features “the domain X has been changed” appeared.

The source code for generating of PFAM domain features is available on github:
https://github.com/Reshetnikoff/m.tuberculosis-research-code

Training of the feature selection algorithms and their performance benchmarking

The python implementation of regularized logistic regression (LR) from the Picasso package [14] was used in our study for SCAD, MCP and L1 regularization and scikit-learn (v. 0.22.1) was used for LR with elastic net regularization. The feature selection procedure by these algorithms was simply the gathering of features having nonzero coefficients on the training set.

We used HHS implementation v. 0.2.0 from the original paper [18]. It was run with three values of the parameter --p1g1_filter: 1, 3 and 5.

The ABESS implementation v. 0.1.0 from the original paper [15] with the default parameters (family='binomial', normalize=0, max_splicing_iter=30, warm.start=False, tune.type='gic', num.threads=24) was used in our study.

For every dataset split the training set was used for the training of the selection algorithms. After the training, a list of features that have non-zero coefficients in the trained model was obtained. Logistic regression (LR) (with solver='saga', max_iter=10000, without regularization) from the “scikit-learn” package (v. 0.22.1) was trained using these features on the same training set . The testing set was used for assessing the prediction quality of the selected features using the ROC-AUC metric and F1 score.

To estimate the statistical significance of the differences in performance of the models trained on the feature sets comprising various aggregated features (see the “Estimating the impact of aggregation” in the main text) the procedure described above was repeated 100 times and Wilcoxon signed-rank test was used from the scipy python module to compute pairwise p-values (Table S3-S8,S10-11). The threshold for statistical significance is 0.0038 which is a standard 0.05 threshold with the Bonferroni correction.

Searching for hyperparameter values

SCAD, MCP and elastic net have two hyperparameters which tune regularization. The first hyperparameter of LR with elastic net regularization from the scikit-learn package is C, the inverse of regularization strength, the second is l1_ratio, which regulates the relative importance of L1 and L2 regularization. SCAD and MCP both have lambda and alpha parameters: to understand their meaning see the papers [16, 17].

We selected three or four arbitrary significantly different values of the second parameter for parametrization of LR regularization functions : l1_ratio equaled 0.25, 0.5 and 0.75 for elastic net and alpha equaled 3, 5, 10 and 15 for SCAD and MCP. For each selected value of the second parameter we found the minimal value of the first parameter (1/C and lambda respectively) for which all features have zero regression coefficients. After that, the interval from the zero value to the found value was divided into 20 equal parts. The value in the middle of each interval and the corresponding value of the second parameter formed pairs of hyperparameter values which were used for estimating the ROC AUC as follows.

For each pair of hyperparameter values the corresponding LR models were trained on the training set. The algorithm contains two steps: the feature selection step and the assessment step of selected features. On the feature selection step the features having nonzero coefficients were selected. On the next assessment step, logistic regression (solver='saga', max_iter=10000, without regularization) was trained on the training set using features selected on the feature selection step and then used for predictions of dependent variables (drug phenotypes) for isolates in the testing set. The pairs of hyperparameters corresponding to the highest roc-auc metrics averaged over five dataset splits were choused. This procedure was applied separately for each drug. Finally, for each drug the best pair of hyperparameter values chosen for each LR regularization function was used for further analysis.

Logistic regression with L1 regularization has only one hyperparameter. It was tuned in the same way as lambda for SCAD and MCP, as it described above.

The original implementation of HHS v. 0.2.0 was used in this study [18]. The method has one hyperparameter - the minimum frequency of mutations that lead to the resistance phenotype. The values of the hyperparameter equal to 1, 3 and 5 were tested. For each hyperparameter value and for each drug HHS algorithm was used for the feature selection. Then, selected features were used for training of the LR model (solver='saga', max_iter=10000, without regularization) on the training set of each dataset split. For each drug, this parameter was selected to achieve the best prediction performance measured as average roc-auc on test sets of dataset splits.

Selection of the final feature set

After the training, a list of features that have non-zero coefficients in each trained model was obtained for each dataset split. For each feature from the lists the number of splits in which this feature was selected was computed (Table S12-S17). We defined a feature to be majorly selected if it was selected in three or more dataset splits (Table 3). For each feature (including domain features), Fisher exact test p-value was computed using the "scipy" python package on the testing set of the first dataset split. This allowed us to evade the overfitting bias of domain aggregation feature binarization: the threshold was obtained on the training set, while the p-value was calculated on the whole dataset. This procedure allowed us to filter by p-value the majorly selected features for the subsequent search for their biological interpretation. Unfortunately, some of the stably selected features were rare enough to never occur in the test set of that particular split, so these features were excluded from this analysis. Finally, for ABESS and HHS the majorly selected features were filtered by Fisher p-value with threshold 0.05. We also report the mean values of coefficients of all selected features from the trained models (Table S12-S17).

Accounting for the population structure

The phylogenetic tree was built with IQ-Tree (v. 2.2.0) using the GTR+G model. Since full genomes are too large the pseudo alignment was used instead of full MSA: all constant positions and indels were excluded. The positions in the genes associated with the drug resistance in the literature (Table S1) were also excluded.

The big tree was pruned for each drug using the "prune" function from the ete3 (v. 3.0) python package [19] keeping only leaves with known phenotype. TreeBreaker v. 1.1 was used to find the clades on each phylogenetic tree with phenotype distribution different from the parental clades. The resulting annotated trees were parsed with ete3 and internal nodes with posterior probability of distribution switch higher than 0.5 were selected.

The geographical locations of isolates were obtained from the NCBI BioSample database using the Entrez module from the biopython (v. 1.79) package [13].

The correspondence of isolates to the TB-Profiler phylogenetic lineages were inferred using the lineage-defining SNPs from the TB-Profiler [20] database (https://github.com/jodyphelan/TBProfiler/tree/master/db/tbdb.barcode.bed version from 2021.01.11). Then for each isolate, the lineages were sorted from higher order lineages to their sublineages and only the deepest sublineage was kept.

The second iteration of ABESS and HHS

We defined a resistant isolate to be explained by ABESS on a given dataset split if logistic regression trained on the training set of this split using the features majorly selected by ABESS classifies this isolate as resistant. The fraction of explained isolates was computed for all isolates in the training set of each dataset split and values obtained for all splits were averaged. The coefficients of the logistic regression models trained on all splits were averaged for each selected feature.

The second iteration was performed on the same dataset splits. For each split all resistant isolates explained on the first iteration were removed. Then random susceptible isolates were removed to preserve the fraction of the resistant isolates in the dataset. All features which were majorly selected for at least one drug with positive coefficients at the first iteration were completely removed from the dataset at the second iteration enforcing ABESS to search for new associations. After the ABESS training feature selection process was repeated: we computed the number of times each feature was selected. Then logistic regression was trained again on the set of majorly selected features, the regression coefficients were averaged and new explained isolates were counted for each drug (Table 4,5 and S31). The same procedure was performed for HHS algorithm (Table S32-S34)

Supplementary figures

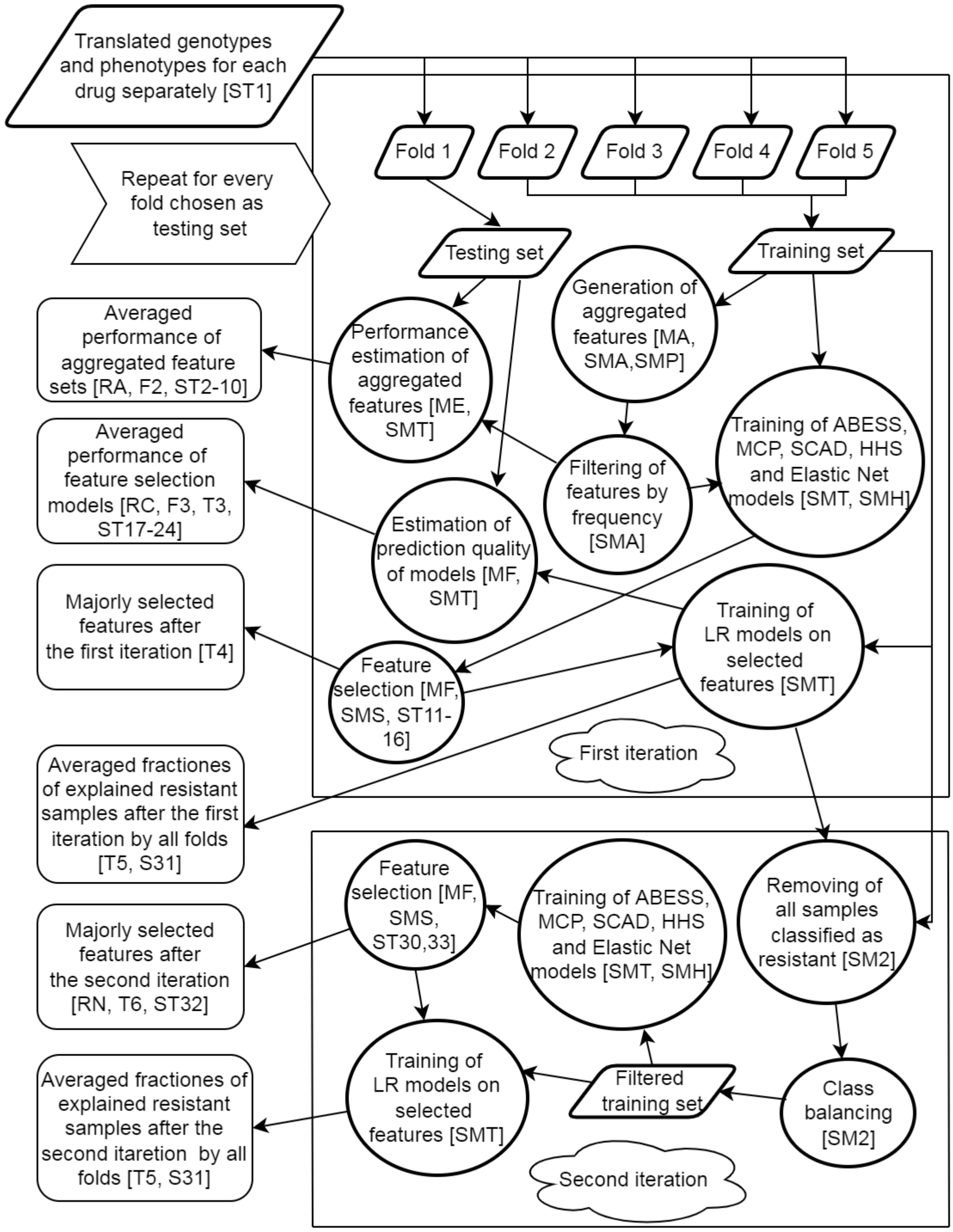

Fig. S1. The main steps of the study. To read about each step see the section listed in the square brackets (i.e. [MA] etc.). In Methods: MA - "Aggregation of mutations", ME - "Estimating the impact of aggregation", MF - "Feature selection and model quality evaluation"; in Supplementary Methods: SMA - "Aggregation of mutations", SMP - "PFAM domains based feature aggregation", SMT - "Training of the feature selection algorithms and their performance benchmarking", SMH - "Searching for hyperparameter values", SMS - "Selection of the final majorly selected feature set", SM2 - "The second iteration of ABESS and HHS"; in Results: RA - "Aggregation of mutations improves the prediction quality", RC -

“Comparison of the feature selection algorithms”, RN - “New associations can be found by repeating the search on the unexplained resistant isolates”. F# means Figure #, T# means Table #, ST# means Supplementary Table #.

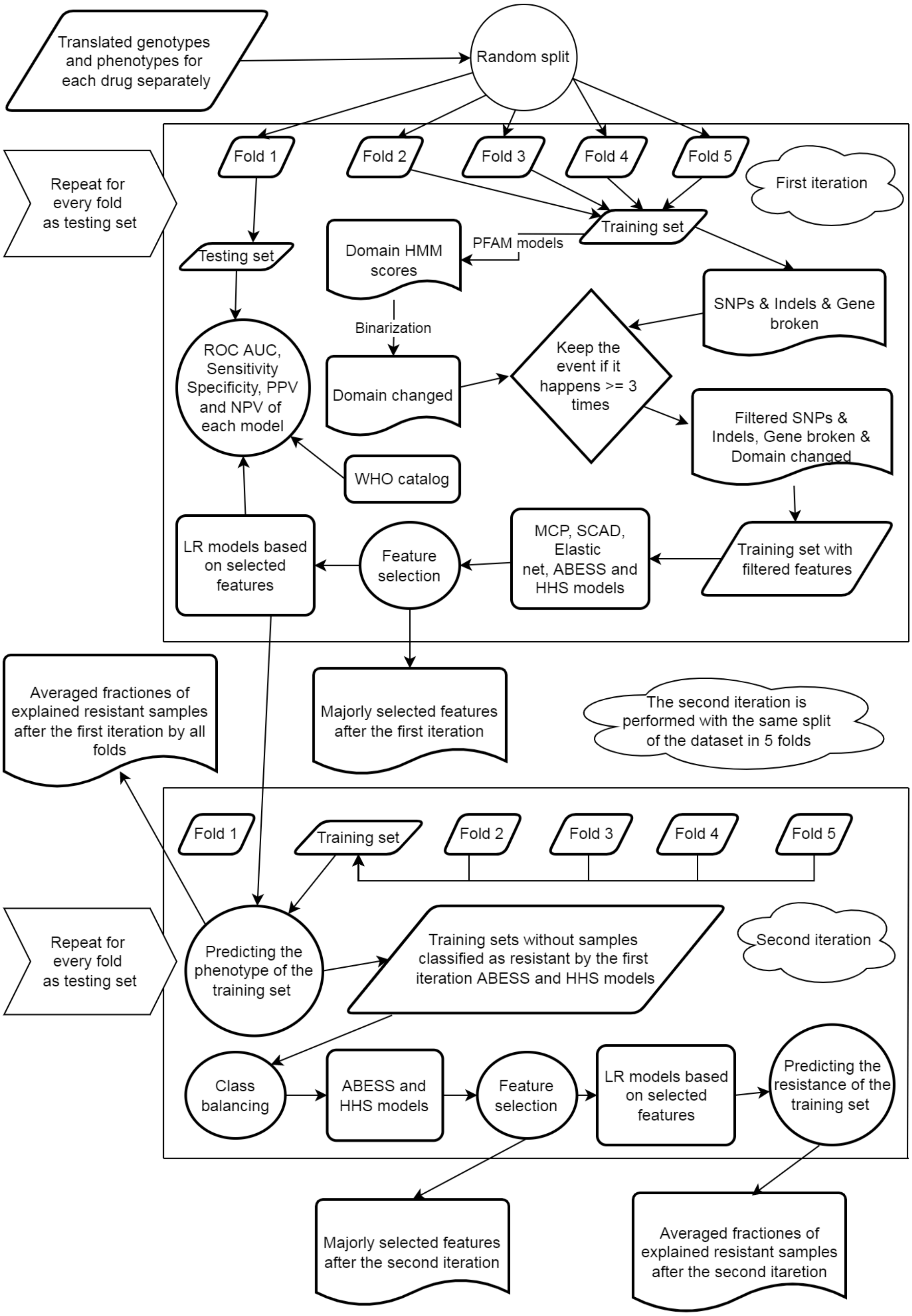

Fig. S2. The detailed schema of the comparison of feature aggregation strategies.

Supplementary tables.

Table S1. Genes associated with resistance to drugs used in tuberculosis treatment, based on previous studies. Genes from the 2021 WHO catalog are marked in bold. The asterisk (*) indicates the *eis* gene, which is present in the WHO catalog but not in the other studies.

Drug name	Classification by line	Drug group	Gene name	References
Rifampicin	First line		***rpoB***, *ponA1*	[21, 22]
Isoniazid			*ahpC*, ***fabG1***, ***inhA***, ***katG***, *ndh*	[9, 21, 22]
Pyrazinamide			***pncA***, *rpsA*, *panD*	[21, 22]
Ethambutol			*embA*, ***embB***, *embC*, *embR*, *iniA*, *iniC*, *manB*, *rmlD*, *ubiA*	[9, 21–23]
Streptomycin		Aminoglycosides	***gidB***, ***rrs***, ***rpsL***	[9, 21, 22]
Kanamycin	Second line		***rrs***, ***eis***	[9, 21, 22]
Amikacin			***rrs***, ***eis****	[9, 21, 22]
Capreomycin			***rrs***, ***tlyA***	[9, 21, 22]
Ofloxacin		Fluoroquinolones	*gyrA*, *gyrB*	[9]
Moxifloxacin			***gyrA***, ***gyrB***	[21, 22]
Ciprofloxacin			*gyrA*, *gyrB*	[9]
Ethionamide		Thiocarbamide derivative	***ethA***, *ethR*, ***inhA***	[21, 22]
Prothionamide			***ethA***, *ethR*, ***inhA***	[21, 22]

Table S2. Experimentally validated phenotypes of all isolates from our dataset with data sources. "S" means susceptible, "R" means resistant, "-" means unknown. https://github.com/Reshetnikoff/m.tuberculosis-research-code/blob/main/Supplementary%20tables/Table%20S2.zip

Table S3. Effect of addition of PFAM domains on the mean ROC AUC of logistic regression estimated by multiple 5 fold cross-validations. The p-value is calculated by the Wilcoxon signed-rank test on 100 random replications. Higher values for each drug and group are marked in bold.

	SNPs & Indels	SNPs & Indels & PFAM	P-value
Rifampicin	**0,9825**	0,9815	7,1E-18
Isoniazid	**0,9759**	0,9756	1,2E-06
Pyrazinamide	0,9439	**0,9507**	3,9E-18
Ethambutol	**0,9579**	0,9523	3,9E-18
Streptomycin	0,9421	**0,9511**	3,9E-18
Kanamycin	**0,9515**	0,9494	1,2E-10
Amikacin	0,9198	**0,9223**	3,2E-14
Capreomycin	0,8769	**0,8845**	4,5E-18
Ofloxacin	**0,9087**	0,9055	1,3E-15
Moxifloxacin	0,8961	0,8956	4,5E-01
Ciprofloxacin	0,9866	0,9856	1,4E-02
Ethionamide	0,8236	**0,8566**	3,9E-18
Prothionamide	0,6976	**0,7227**	5,0E-18
First line	0,9605	**0,9622**	3,9E-18
Aminoglycosides	0,9226	**0,9268**	3,9E-18
Fluoroquinolones	**0,9304**	0,9289	1,3E-12
Second line	0,8826	**0,8903**	3,9E-18
All drugs	0,9126	**0,9179**	3,9E-18

Table S4. Effect of addition of “Gene broken” features on the mean ROC AUC of logistic regression estimated by multiple 5 fold cross-validations. The p-value is calculated by the Wilcoxon signed-rank test on 100 random replications. Higher values for each drug and group are marked in bold.

	SNPs & Indels	SNPs & Indels & Broken genes	P-value
Rifampicin	**0,9825**	0,9822	4,3E-14
Isoniazid	**0,9759**	0,9757	1,1E-11
Pyrazinamide	0,9439	**0,9450**	3,6E-16
Ethambutol	0,9579	**0,9584**	3,4E-15
Streptomycin	0,9421	**0,9427**	1,5E-12
Kanamycin	0,9515	**0,9524**	5,8E-09
Amikacin	0,9198	**0,9210**	5,1E-11
Capreomycin	**0,8769**	0,8738	1,5E-17
Ofloxacin	0,9087	**0,9091**	2,7E-03
Moxifloxacin	**0,8961**	0,8946	1,6E-08
Ciprofloxacin	0,9866	**0,9868**	8,2E-03
Ethionamide	0,8236	**0,8364**	3,9E-18

Prothionamide	0,6976	**0,6997**	3,9E-04
First line	0,9605	**0,9608**	6,9E-17
Aminoglycosides	0,9226	0,9225	2,1E-01
Fluoroquinolones	**0,9304**	0,9302	3,5E-03
Second line	0,8826	**0,8843**	3,5E-17
All drugs	0,9126	**0,9137**	1,4E-17

Table S5. Effect of addition of gene aggregation on the mean ROC AUC of logistic regression estimated by multiple 5 fold cross-validations. The p-value is calculated by the Wilcoxon signed-rank test on 100 random replications. Higher values for each drug and group are marked in bold.

	**SNPs & Indels**	**SNPs & Indels & gene aggregation**	**P-value**
Rifampicin	**0,9825**	0,9810	3,9E-18
Isoniazid	**0,9759**	0,9748	1,7E-17
Pyrazinamide	0,9439	**0,9444**	5,4E-04
Ethambutol	**0,9579**	0,9524	3,9E-18
Streptomycin	**0,9421**	0,9407	1,2E-14
Kanamycin	**0,9515**	0,9460	4,5E-18
Amikacin	**0,9198**	0,9176	2,2E-14
Capreomycin	**0,8769**	0,8756	1,6E-05
Ofloxacin	0,9087	**0,9096**	7,9E-05
Moxifloxacin	0,8961	**0,9024**	5,6E-18
Ciprofloxacin	**0,9866**	0,9861	6,0E-02
Ethionamide	0,8236	**0,8431**	3,9E-18
Prothionamide	**0,6976**	0,6891	1,7E-14
First line	**0,9605**	0,9587	3,9E-18
Aminoglycosides	**0,9226**	0,9200	3,9E-18
Fluoroquinolones	0,9304	**0,9327**	2,3E-17
Second line	0,8826	**0,8837**	6,2E-11
All drugs	0,9126	0,9125	5,3E-01

Table S6. Effect of addition of PFAM domains on the mean F1 score of logistic regression estimated by multiple 5 fold cross-validations. The p-value is calculated by the Wilcoxon signed-rank test on 100 random replications. Higher values for each drug and group are marked in bold.

	SNPs & Indels	SNPs & Indels & PFAM	P-value
Rifampicin	0,9348	**0,9379**	5,9E-18
Isoniazid	0,9322	**0,9362**	3,9E-18
Pyrazinamide	0,7153	**0,7607**	3,9E-18
Ethambutol	**0,7580**	0,7508	4,1E-18
Streptomycin	0,8577	**0,8711**	3,9E-18
Kanamycin	**0,8736**	0,8679	9,9E-12
Amikacin	0,8298	**0,8342**	1,1E-09
Capreomycin	**0,7450**	0,7405	6,5E-07
Ofloxacin	**0,7850**	0,7757	5,8E-16
Moxifloxacin	**0,7088**	0,7017	4,0E-07
Ciprofloxacin	0,8926	**0,9046**	8,6E-13
Ethionamide	0,6684	**0,7040**	3,9E-18
Prothionamide	0,5555	**0,5937**	4,5E-18
First line	0,8396	**0,8513**	3,9E-18
Aminoglycosides	0,8265	**0,8285**	1,3E-07
Fluoroquinolones	0,7955	0,7940	5,3E-02
Second line	**0,7573**	0,7653	6,5E-18
All drugs	**0,7890**	0,7984	3,9E-18

Table S7. Effect of addition of “Gene broken” features on the mean F1 score of logistic regression estimated by multiple 5 fold cross-validations. The p-value is calculated by the Wilcoxon signed-rank test on 100 random replications. Higher values for each drug and group are marked in bold.

	SNPs & Indels	SNPs & Indels & Broken gene	P-value
Rifampicin	**0,9348**	0,9342	5,3E-09
Isoniazid	0,9322	0,9324	1,2E-02
Pyrazinamide	0,7153	**0,7192**	6,5E-10
Ethambutol	**0,7580**	0,7547	5,9E-13
Streptomycin	0,8577	**0,8585**	2,8E-03
Kanamycin	0,8736	**0,8766**	5,7E-07
Amikacin	0,8298	0,8306	5,3E-02
Capreomycin	0,7450	0,7439	3,5E-02
Ofloxacin	**0,7850**	0,7821	1,3E-08
Moxifloxacin	**0,7088**	0,7047	9,3E-05
Ciprofloxacin	0,8926	0,8917	1,2E-01

Ethionamide	**0,6684**	0,6925	4,8E-18
Prothionamide	0,5555	0,5529	1,2E-01
First line	0,8396	0,8398	1,7E-01
Aminoglycosides	0,8265	**0,8274**	1,8E-03
Fluoroquinolones	**0,7955**	0,7928	1,5E-08
Second line	0,7573	**0,7594**	4,6E-09
All drugs	0,7890	**0,7903**	8,3E-09

Table S8. Effect of addition of gene aggregation on the mean F1 score of logistic regression estimated by multiple 5 fold cross-validations. The p-value is calculated by the Wilcoxon signed-rank test on 100 random replications. Higher values for each drug and group are marked in bold.

	**SNPs & Indels**	**SNPs & Indels & gene aggregation**	**P-value**
Rifampicin	**0,9348**	0,9340	7,2E-06
Isoniazid	0,9322	0,9320	2,6E-01
Pyrazinamide	0,7153	**0,7357**	3,9E-18
Ethambutol	**0,7580**	0,7499	4,3E-18
Streptomycin	0,8577	**0,8596**	2,4E-07
Kanamycin	**0,8736**	0,8714	8,8E-04
Amikacin	0,8298	**0,8317**	1,6E-04
Capreomycin	**0,7450**	0,7414	5,7E-06
Ofloxacin	**0,7850**	0,7765	3,3E-17
Moxifloxacin	0,7088	**0,7131**	6,8E-05
Ciprofloxacin	0,8926	**0,8966**	8,6E-04
Ethionamide	0,6684	**0,6910**	6,9E-18
Prothionamide	0,5555	0,5553	8,6E-01
First line	0,8396	**0,8422**	7,7E-16
Aminoglycosides	0,8265	0,8260	1,3E-01
Fluoroquinolones	0,7955	0,7954	6,8E-01
Second line	0,7573	**0,7596**	8,0E-08
All drugs	0,7890	**0,7914**	1,5E-13

Table S9. The unbiased sample variance of ROC AUC of logistic regression with L1 regularization trained on feature sets containing different aggregated features.

	SNPs & Indels	SNPs & Indels & PFAM	SNPs & Indels & broken genes	SNPs & Indels & gene aggregation
Rifampicin	3,9E-07	6,1E-07	4,6E-07	6,0E-07
Isoniazid	4,8E-07	6,8E-07	5,2E-07	6,1E-07
Pyrazinamide	2,3E-06	2,3E-06	2,7E-06	2,7E-06
Ethambutol	6,5E-07	1,2E-06	5,9E-07	9,3E-07
Streptomycin	2,7E-06	2,2E-06	3,0E-06	3,3E-06
Kanamycin	1,3E-05	8,9E-06	1,2E-05	1,3E-05
Amikacin	1,1E-05	1,2E-05	1,2E-05	1,3E-05
Capreomycin	2,2E-05	2,6E-05	2,0E-05	2,3E-05
Ofloxacin	1,1E-05	1,3E-05	1,2E-05	9,7E-06
Moxifloxacin	2,0E-05	2,9E-05	2,1E-05	2,3E-05
Ciprofloxacin	1,2E-05	2,4E-05	1,2E-05	1,3E-05
Ethionamide	4,4E-05	5,9E-05	5,4E-05	5,3E-05
Prothionamide	1,5E-04	1,5E-04	1,6E-04	1,8E-04

Table S10. Comparison of aggregation with PFAM domains and gene aggregation. The mean ROC AUC of logistic regression estimated by multiple 5 fold cross-validations. The p-value is calculated by the Wilcoxon signed-rank test on 100 random replications. Higher values for each drug and group are marked in bold.

	SNPs & Indels & PFAM	SNPs & Indels & gene aggregation	P-value
Rifampicin	**0,9815**	0,9810	3,4E-08
Isoniazid	**0,9756**	0,9748	1,2E-13
Pyrazinamide	**0,9507**	0,9444	3,9E-18
Ethambutol	0,9523	0,9524	2,3E-01
Streptomycin	**0,9511**	0,9407	3,9E-18
Kanamycin	**0,9494**	0,9460	1,2E-15
Amikacin	**0,9223**	0,9176	4,1E-18
Capreomycin	**0,8845**	0,8756	3,9E-18
Ofloxacin	0,9055	**0,9096**	2,3E-17
Moxifloxacin	0,8956	**0,9024**	3,8E-17
Ciprofloxacin	0,9856	0,9861	3,0E-01
Ethionamide	**0,8566**	0,8431	5,8E-18
Prothionamide	**0,7227**	0,6891	3,9E-18
First line	**0,9622**	0,9587	3,9E-18
Aminoglycosides	**0,9268**	0,9200	3,9E-18

Fluoroquinolones	0,9289	**0,9327**	4,7E-18
Second line	**0,8903**	0,8837	3,9E-18
All drugs	**0,9179**	0,9125	3,9E-18

Table S11. Comparison of aggregation with PFAM domains and gene aggregation. The mean F1 score of logistic regression estimated by multiple 5 fold cross-validations. The p-value is calculated by the Wilcoxon signed-rank test on 100 random replications. Higher values for each drug and group are marked in bold.

	**SNPs & Indels & PFAM**	**SNPs & Indels & gene aggregation**	**P-value**
Rifampicin	**0,9379**	0,9340	4,7E-18
Isoniazid	**0,9362**	0,9320	3,9E-18
Pyrazinamide	**0,7607**	0,7357	3,9E-18
Ethambutol	0,7508	0,7499	2,6E-02
Streptomycin	**0,8711**	0,8596	3,9E-18
Kanamycin	0,8679	**0,8714**	2,0E-05
Amikacin	**0,8342**	0,8317	8,3E-05
Capreomycin	0,7405	0,7414	2,6E-01
Ofloxacin	0,7757	0,7765	4,2E-01
Moxifloxacin	0,7017	**0,7131**	4,5E-11
Ciprofloxacin	**0,9046**	0,8966	5,4E-08
Ethionamide	**0,7040**	0,6910	4,4E-12
Prothionamide	**0,5937**	0,5553	1,4E-17
First line	**0,8513**	0,8422	3,9E-18
Aminoglycosides	**0,8285**	0,8260	2,6E-09
Fluoroquinolones	0,7940	0,7954	6,4E-02
Second line	**0,7653**	0,7596	5,9E-16
All drugs	**0,7984**	0,7914	4,1E-18

Table S12. The features selected by ABESS. The features whose values fully coincide on the given dataset split were unified and were treated as one feature during the selection process. All features were enumerated for each drug and the same IDs were assigned to the features constituting unified ones.

https://github.com/Reshetnikoff/m.tuberculosis-research-code/blob/main/Supplementary%20tables/Table%20S12.tsv

Table S13. The features selected by HHS. The features whose values fully coincide on the given dataset split were unified and were treated as one feature during the selection

process. All features were enumerated for each drug and the same IDs were assigned to the features constituting unified ones.

https://github.com/Reshetnikoff/m.tuberculosis-research-code/blob/main/Supplementary%20tables/Table%20S13.tsv

Table S14. The features selected by logistic regression with SCAD regularization. The features whose values fully coincide on the given dataset split were unified and were treated as one feature during the selection process. All features were enumerated for each drug and the same IDs were assigned to the features constituting unified ones.

https://github.com/Reshetnikoff/m.tuberculosis-research-code/blob/main/Supplementary%20tables/Table%20S14.tsv

Table S15. The features selected by logistic regression with MCP regularization. The features whose values fully coincide on the given dataset split were unified and were treated as one feature during the selection process. All features were enumerated for each drug and the same IDs were assigned to the features constituting unified ones.

https://github.com/Reshetnikoff/m.tuberculosis-research-code/blob/main/Supplementary%20tables/Table%20S15.tsv

Table S16. The features selected by logistic regression with L1 regularization. The features whose values fully coincide on the given dataset split were unified and were treated as one feature during the selection process. All features were enumerated for each drug and the same IDs were assigned to the features constituting unified ones.

https://github.com/Reshetnikoff/m.tuberculosis-research-code/blob/main/Supplementary%20tables/Table%20S16.tsv

Table S17. The features selected by logistic regression with elastic net regularization. The features whose values fully coincide on the given dataset split were unified and were treated as one feature during the selection process. All features were enumerated for each drug and the same IDs were assigned to the features constituting unified ones.

https://github.com/Reshetnikoff/m.tuberculosis-research-code/blob/main/Supplementary%20tables/Table%20S17.tsv

Table S18. The quality metrics of ABESS obtained by 5-fold cross-validation.

**Drug name**	**AUC**	**Sensitivity**	**Specivity**	**NPV**	**PPV**
Kanamycin	0,92	0,77	0,98	0,91	0,94
Amikacin	0,86	0,72	0,99	0,93	0,96
Streptomycin	0,92	0,75	0,96	0,88	0,92
Ofloxacin	0,80	0,68	0,93	0,90	0,75

Moxifloxacin	0,82	0,56	0,94	0,89	0,72
Isoniazid	0,96	0,90	0,98	0,96	0,96
Rifampicin	0,97	0,90	0,99	0,97	0,96
Ethambutol	0,93	0,75	0,94	0,95	0,69
Pyrazinamide	0,90	0,62	0,97	0,95	0,71
Capreomycin	0,84	0,64	0,96	0,91	0,82
Ethionamide	0,78	0,60	0,89	0,77	0,78
Prothionamide	0,59	0,33	0,85	0,65	0,61
Ciprofloxacin	0,88	0,57	0,97	0,89	0,85

Table S19. The quality metrics of HHS obtained by 5-fold cross-validation.

**Drug name**	**AUC**	**Sensitivity**	**Specivity**	**NPV**	**PPV**
Amikacin	0,86	0,72	0,99	0,93	0,96
Ofloxacin	0,79	0,58	0,98	0,88	0,91
Pyrazinamide	0,66	0,20	0,99	0,90	0,83
Rifampicin	0,64	0,24	1,00	0,80	0,96
Capreomycin	0,82	0,66	0,96	0,91	0,81
Ciprofloxacin	0,79	0,53	0,98	0,88	0,89
Ethambutol	0,62	0,24	0,99	0,88	0,73
Ethionamide	0,69	0,54	0,85	0,74	0,69
Isoniazid	0,59	0,18	0,99	0,74	0,93
Kanamycin	0,88	0,76	0,98	0,91	0,95
Moxifloxacin	0,75	0,48	0,97	0,87	0,81
Prothionamide	0,57	0,18	0,95	0,63	0,76
Streptomycin	0,56	0,14	0,97	0,67	0,73

Table S20. The quality metrics of logistic regression with SCAD regularization obtained by 5-fold cross-validation.

**Drug name**	**AUC**	**Sensitivity**	**Specivity**	**NPV**	**PPV**
Kanamycin	0,95	0,83	0,96	0,93	0,89
Amikacin	0,92	0,75	0,98	0,93	0,92
Streptomycin	0,95	0,84	0,94	0,91	0,88
Ofloxacin	0,90	0,70	0,95	0,91	0,81
Moxifloxacin	0,90	0,62	0,95	0,90	0,77
Isoniazid	0,98	0,90	0,99	0,96	0,97
Rifampicin	0,98	0,91	0,99	0,97	0,97
Ethambutol	0,95	0,75	0,96	0,96	0,75

Pyrazinamide	0,95	0,74	0,97	0,96	0,80
Capreomycin	0,88	0,65	0,96	0,91	0,80
Ethionamide	0,86	0,69	0,86	0,81	0,77
Prothionamide	0,72	0,58	0,74	0,72	0,61
Ciprofloxacin	0,98	0,90	0,99	0,97	0,96

Table S21. The quality metrics of logistic regression with MCP regularization obtained by 5-fold cross-validation.

**Drug name**	**AUC**	**Sensitivity**	**Specivity**	**NPV**	**PPV**
Kanamycin	0,95	0,83	0,96	0,93	0,89
Amikacin	0,92	0,75	0,98	0,93	0,92
Streptomycin	0,95	0,84	0,94	0,91	0,88
Ofloxacin	0,90	0,70	0,95	0,91	0,81
Moxifloxacin	0,90	0,62	0,95	0,90	0,77
Isoniazid	0,98	0,90	0,99	0,96	0,97
Rifampicin	0,98	0,91	0,99	0,97	0,97
Ethambutol	0,95	0,75	0,96	0,96	0,75
Pyrazinamide	0,95	0,74	0,97	0,96	0,80
Capreomycin	0,88	0,65	0,96	0,91	0,80
Ethionamide	0,86	0,69	0,86	0,81	0,77
Prothionamide	0,72	0,58	0,74	0,72	0,61
Ciprofloxacin	0,98	0,90	0,99	0,97	0,96

Table S22. The quality metrics of logistic regression with L1 regularization obtained by 5-fold cross-validation.

**Drug name**	**AUC**	**Sensitivity**	**Specivity**	**NPV**	**PPV**
Kanamycin	0,96	0,83	0,95	0,93	0,88
Amikacin	0,92	0,75	0,98	0,93	0,92
Streptomycin	0,95	0,84	0,94	0,92	0,88
Ofloxacin	0,90	0,70	0,95	0,91	0,82
Moxifloxacin	0,90	0,62	0,95	0,90	0,78
Isoniazid	0,98	0,91	0,99	0,96	0,97
Rifampicin	0,98	0,91	0,99	0,97	0,97
Ethambutol	0,95	0,75	0,96	0,96	0,77
Pyrazinamide	0,95	0,73	0,97	0,96	0,79
Capreomycin	0,88	0,68	0,96	0,92	0,82
Ethionamide	0,86	0,68	0,86	0,80	0,76

Prothionamide	0,72	0,58	0,74	0,72	0,62
Ciprofloxacin	0,98	0,91	0,99	0,98	0,96

Table S23. The quality metrics of logistic regression with elastic net regularization obtained by 5-fold cross-validation.

	**AUC**	**Sensitivity**	**Specivity**	**NPV**	**PPV**
Rifampicin	0,98	0,89	0,99	0,96	0,96
Isoniazid	0,97	0,90	0,99	0,96	0,97
Pyrazinamide	0,95	0,73	0,97	0,96	0,79
Ethambutol	0,95	0,73	0,95	0,95	0,73
Streptomycin	0,95	0,83	0,94	0,91	0,88
Kanamycin	0,95	0,81	0,96	0,93	0,89
Amikacin	0,92	0,74	0,99	0,93	0,95
Capreomycin	0,87	0,65	0,97	0,91	0,84
Ofloxacin	0,90	0,69	0,96	0,91	0,86
Moxifloxacin	0,90	0,62	0,95	0,90	0,78
Ciprofloxacin	0,98	0,90	0,99	0,97	0,97
Ethionamide	0,87	0,65	0,86	0,79	0,76
Prothionamide	0,71	0,55	0,74	0,70	0,60

Table S24. The quality metrics of the dictionary method based on WHO catalog.

**Drug name**	**AUC**	**Sensitivity**	**Specivity**	**NPV**	**PPV**
Amikacin	0,86	0,74	0,98	0,93	0,91
Capreomycin	0,81	0,66	0,96	0,92	0,81
Ethambutol	0,89	0,84	0,93	0,97	0,69
Ethionamide	0,73	0,61	0,84	0,77	0,72
Isoniazid	0,93	0,88	0,99	0,95	0,98
Kanamycin	0,91	0,83	0,98	0,94	0,94
Moxifloxacin	0,81	0,69	0,94	0,92	0,76
Pyrazinamide	0,77	0,56	0,98	0,94	0,82
Rifampicin	0,95	0,93	0,98	0,98	0,95
Streptomycin	0,87	0,78	0,97	0,89	0,92
Prothionamide	0,60	0,24	0,95	0,76	0,64

Table S25. The comparison of stability of feature selection by different methods. The metrics are: the number of selected features averaged by dataset splits, the average fraction of majorly selected features from all features selected in each data split and the mean number of dataset splits in which the majorly selected features were selected. The maximal value of the metric for each drug is marked in bold.

https://github.com/Reshetnikoff/m.tuberculosis-research-code/blob/main/Supplementary%20tables/Table%20S25.tsv

Table S26. The clades selected by TreeBreaker for each drug and their composition. Each isolate ID from the clades is presented in the last column with corresponding ID of the internal node from the second column which forms the clade. Some clades contain the other clades as subclades, so some isolate IDs are repeated for the same drug.

https://github.com/Reshetnikoff/m.tuberculosis-research-code/blob/main/Supplementary%20tables/Table%20S26.zip

Table S27. The descriptive statistics of TreeBreaker clades. For each drug the number of clades detected by TreeBreaker, the number of clades associated with location from the detected ones and the number of clades associated with lineages from the detected ones are presented.

Drug	TreeBreaker clades	Associated with location	Associated with lineage
Ciprofloxacin	18	0	8
Prothionamide	1	0	1
Ethionamide	12	1	10
Kanamycin	24	3	18
Moxifloxacin	10	3	6
Amikacin	26	10	21
Capreomycin	11	3	8
Ofloxacin	16	4	14
Streptomycin	47	18	36
Pyrazinamide	102	15	58
Ethambutol	92	20	64
Isoniazid	157	34	125
Rifampicin	163	28	111

Table S28. Geographic locations and TBDB lineages of isolates.

https://github.com/Reshetnikoff/m.tuberculosis-research-code/blob/main/Supplementary%20tables/Table%20S28.zip

Table S29. Locations and lineage characteristics of TreeBreaked clades. For each drug and each node_id of the clade root the number of resistant and susceptible isolates and the number of isolates for which the sampling location is known are presented. The last two columns present Bonferroni-corrected p-values of association of each clade with locations and lineages.

https://github.com/Reshetnikoff/m.tuberculosis-research-code/blob/main/Supplementary%20tables/Table%20S29.zip

Table S30. The features which were majorly selected by the ABESS algorithm trained using the feature set extended with features generated by TreeBreaker. All features were enumerated for each drug and the same IDs were assigned to the features constituting unified ones.

https://github.com/Reshetnikoff/m.tuberculosis-research-code/blob/main/Supplementary%20tables/Table%20S30.xlsx

Table S31. The features selected by the second iteration of ABESS. The features whose values fully coincide on the given dataset split were unified and were treated as one feature during the selection process. All features were enumerated for each drug and the same IDs were assigned to the features constituting unified ones.

https://github.com/Reshetnikoff/m.tuberculosis-research-code/blob/main/Supplementary%20tables/Table%20S31.zip

Table S32. The statistics of explained resistant isolates by the first and the second iteration of the HHS algorithm. We defined a resistant isolate to be explained by HHS on a given dataset split if logistic regression trained on the training set of this split using the features majorly selected by HHS classifies this isolate as resistant. All the numbers were averaged by splits. In case of increase of the fraction of explained isolates for the given drug the values after the first and the second iteration are marked in bold.

**Drug name**	**Number of resistant isolates**	**Number of isolates participated in the first ABESS iteration**	**Fraction of resistant isolates explained by the first ABESS iteration**	**Number of resistant isolates unexplained after the first ABESS iteration**	**Number of isolates participated in the second ABESS iteration**	**Fraction of resistant isolates explained by the first and the second ABESS iteration**	**Number of resistant isolates unexplained after the second ABESS iteration**
Rifampicin	2277	9319	**26,43%**	1675	6856	**88,19%**	269
Isoniazid	2754	9252	**17,68%**	2267	7616	**89,46%**	290
Pyrazinamide	884	7334	**33,26%**	590	4894	**81,95%**	160
Ethambutol	1223	8143	**4,86%**	1164	7747	**64,76%**	431
Streptomycin	1344	3769	**9,48%**	1217	3411	**25,64%**	999
Kanamycin	275	961	**81,98%**	50	173	**86,34%**	38
Amikacin	322	1482	**73,45%**	86	393	**74,69%**	82

Capreomycin	323	1547	**67,26%**	106	506	**69,06%**	100
Ofloxacin	376	1559	**56,38%**	164	679	**68,14%**	120
Moxifloxacin	206	964	**50,88%**	101	473	**53,31%**	96
Ciprofloxacin	81	366	**48,76%**	41	187	**65,35%**	28
Ethionamide	222	553	**63,54%**	81	201	**73,10%**	60
Prothionamide	144	351	**19,44%**	116	282	**36,81%**	91

Table S33. The additional mutations majorly selected by HHS on the second iteration. The names of genes which were associated with resistance to the given drug in previous studies (Table 1) assigned to the right drugs are marked in bold and underscored otherwise.

Drug name	Gene	Position	Type of event	From	To	Feature ID	Number of splits in which the feature was selected
Rifampicin	***rpoB***	435	SNV	D	G	0	5
Rifampicin	***rpoB***	445	SNV	H	S	1	4
Rifampicin	***rpoB***	432	SNV	Q	K	2	4
Rifampicin	***rpoB***	450	SNV	S	L	3	4
Rifampicin	***rpoB***	433	Insertion	-	F	4	4
Rifampicin	*eccC2*	905	SNV	M	T	5	4
Rifampicin	*dnaJ2*	-	PF00226 is changed	-	-	6	3
Rifampicin	*nmtR*	-47	SNV	T	C	7	3
Rifampicin	*mshD*	71	SNV	R	W	8	3
Rifampicin	*Rv3916c*	-62	SNV	C	T	9	3
Rifampicin	***rpoB***	445	SNV	H	P	10	3
Rifampicin	*lppN*	-46	SNV	A	G	11	3
Rifampicin	*gmhB*	-	Broken	-	-	12	3
Rifampicin	*suhB*	263	Deletion	V	-	13	3
Isoniazid	***katG***	-	PF00141 is changed	-	-	0	3
Pyrazinamide	***pncA***	-	PF00857 is changed	-	-	0	5
Ethambutol	***embB***	1002	SNV	H	R	0	5
Ethambutol	***embB***	306	SNV	M	L	1	5
Ethambutol	***embB***	306	SNV	M	V	2	5
Ethambutol	***embB***	306	SNV	M	I	3	3
Streptomycin	***rrs***	514	SNV	T	G	0	4
Streptomycin	***rpsL***	88	SNV	K	R	1	3

Streptomycin	*Rv1465*	-	PF01592 is changed	-	-	2	3
Kanamycin	*rpoC*	-	PF00623 is changed	-	-	0	3
Amikacin	*Rv2168c*	-52	Insertion	G	ATATCGCCGACAGCCGGTGACGACCCGGCTG	0	3
Amikacin	*mbtA*	-	PF00501 is changed	-	-	1	3
Capreomycin	*nanT*	-	PF07690 is changed	-	-	0	3
Capreomycin	*Rv2752c*	-	PF12706 is changed	-	-	1	3
Capreomycin	*Rv1986*	-	PF01810 is changed	-	-	2	3
Ofloxacin	*ald*	-	PF01262 is changed	-	-	0	5
Ofloxacin	*embA*	-16	SNV	G	A	1	5
Ofloxacin	***gyrB***	-	PF01751 is changed	-	-	2	4
Ofloxacin	*hbhA*	161	Deletion	KK	-	3	3
Moxifloxacin	*embA*	-12	SNV	G	A	0	4
Moxifloxacin	*mdh*	-	PF02866 is changed	-	-	1	3
Moxifloxacin	*rpoC*	527	SNV	L	V	2	3
Moxifloxacin	*rocA*	-	PF00171 is changed	-	-	3	3
Ciprofloxacin	*rpoC*	-	PF00623 is changed	-	-	0	3
Ethionamide	***ethA***	-	PF00743 is changed	-	-	0	4
Ethionamide	*gnd1*	-	PF00393 is changed	-	-	1	3
Prothionamide	*nrdZ*	-	PF02867 is changed	-	-	0	4
Prothionamide	*Rv0566c*	-	PF04461 is changed	-	-	1	3
Prothionamide	*ndh*	-	PF07992 is changed	-	-	2	3

Table S34. The features selected by the second iteration of HHS. All features were enumerated for each drug and the same IDs were assigned to the features constituting unified ones.

https://github.com/Reshetnikoff/m.tuberculosis-research-code/blob/main/Supplementary%20tables/Table%20S34.zip

References

1. **Zhang H, Li D, Zhao L, Fleming J, Lin N, *et al.*** Genome sequencing of 161 Mycobacterium tuberculosis isolates from China identifies genes and intergenic regions associated with drug resistance. *Nat Genet* 2013;45:1255–1260.
2. **Farhat MR, Shapiro BJ, Kieser KJ, Sultana R, Jacobson KR, *et al.*** Genomic analysis identifies targets of convergent positive selection in drug-resistant Mycobacterium tuberculosis. *Nat Genet* 2013;45:1183–1189.
3. **Coll F, Phelan J, Hill-Cawthorne GA, Nair MB, Mallard K, *et al.*** Genome-wide analysis of multi- and extensively drug-resistant Mycobacterium tuberculosis. *Nat Genet* 2018;50:307–316.
4. **Yang C, Luo T, Shen X, Wu J, Gan M, *et al.*** Transmission of multidrug-resistant Mycobacterium tuberculosis in Shanghai, China: a retrospective observational study using whole-genome sequencing and epidemiological investigation. *Lancet Infect Dis* 2017;17:275–284.
5. **Farhat MR, Freschi L, Calderon R, Ioerger T, Snyder M, *et al.*** GWAS for quantitative resistance phenotypes in Mycobacterium tuberculosis reveals resistance genes and regulatory regions. *Nat Commun* 2019;10:2128.
6. **Kouchaki S, Yang Y, Walker TM, Sarah Walker A, Wilson DJ, *et al.*** Application of machine learning techniques to tuberculosis drug resistance analysis. *Bioinformatics* 2019;35:2276–2282.
7. **Casali N, Nikolayevskyy V, Balabanova Y, Harris SR, Ignatyeva O, *et al.*** Evolution and transmission of drug-resistant tuberculosis in a Russian population. *Nat Genet* 2014;46:279–286.
8. **Pankhurst LJ, del Ojo Elias C, Votintseva AA, Walker TM, Cole K, *et al.*** Rapid, comprehensive, and affordable mycobacterial diagnosis with whole-genome sequencing: a prospective study. *Lancet Respir Med* 2016;4:49–58.
9. **Walker TM, Kohl TA, Omar SV, Hedge J, Del Ojo Elias C, *et al.*** Whole-genome sequencing for prediction of Mycobacterium tuberculosis drug susceptibility and resistance: a retrospective cohort study. *Lancet Infect Dis*. Epub ahead of print June 2015. DOI: 10.1016/S1473-3099(15)00062-6.
10. **Johnsen CH, Clausen PTLC, Aarestrup FM, Lund O**. Improved Resistance Prediction in Mycobacterium tuberculosis by Better Handling of Insertions and Deletions, Premature Stop Codons, and Filtering of Non-informative Sites. *Front Microbiol*;10. Epub ahead of print 2019. DOI: 10.3389/fmicb.2019.02464.
11. **Van der Auwera GA, Carneiro MO, Hartl C, Poplin R, del Angel G, *et al.*** From FastQ Data to High-Confidence Variant Calls: The Genome Analysis Toolkit Best Practices Pipeline. *Curr Protoc Bioinforma* 2013;43:11.10.1-11.10.33.
12. **Camacho C, Coulouris G, Avagyan V, Ma N, Papadopoulos J, *et al.*** BLAST+: architecture and applications. *BMC Bioinformatics* 2009;10:421.
13. **Cock PJA, Antao T, Chang JT, Chapman BA, Cox CJ, *et al.*** Biopython: freely available Python tools for computational molecular biology and bioinformatics. *Bioinformatics* 2009;25:1422–1423.
14. **Ge J, Li X, Jiang H, Liu H, Zhang T, *et al.*** Picasso: A Sparse Learning Library for High

Dimensional Data Analysis in R and Python. *J Mach Learn Res* 2019;20:1–5.
15. **Zhu J, Wen C, Zhu J, Zhang H, Wang X**. A polynomial algorithm for best-subset selection problem. *Proc Natl Acad Sci* 2020;117:33117.
16. **Fan J, Li R**. Variable Selection via Nonconcave Penalized Likelihood and its Oracle Properties. *J Am Stat Assoc* 2001;96:1348–1360.
17. **Zhang C-H**. Nearly unbiased variable selection under minimax concave penalty. *Ann Stat* 2010;38:894–942.
18. **Libiseller-Egger J, Phelan J, Campino S, Mohareb F, Clark TG**. Robust detection of point mutations involved in multidrug-resistant Mycobacterium tuberculosis in the presence of co-occurrent resistance markers. *PLOS Comput Biol* 2020;16:e1008518.
19. **Huerta-Cepas J, Serra F, Bork P**. ETE 3: Reconstruction, Analysis, and Visualization of Phylogenomic Data. *Mol Biol Evol* 2016;33:1635–1638.
20. **Phelan JE, O'Sullivan DM, Machado D, Ramos J, Oppong YEA, *et al.*** Integrating informatics tools and portable sequencing technology for rapid detection of resistance to anti-tuberculous drugs. *Genome Med* 2019;11:41.
21. **Walker TM, Miotto P, Köser CU, Fowler PW, Knaggs J, *et al.*** The 2021 WHO catalogue of Mycobacterium tuberculosis complex mutations associated with drug resistance: a genotypic analysis. *Lancet Microbe* 2022;3:e265–e273.
22. **Cohen KA, Manson AL, Desjardins CA, Abeel T, Earl AM**. Deciphering drug resistance in Mycobacterium tuberculosis using whole-genome sequencing: progress, promise, and challenges. *Genome Med*;11. Epub ahead of print December 2019. DOI: 10.1186/s13073-019-0660-8.
23. **Zhang L, Zhao Y, Gao Y, Wu L, Gao R, *et al.*** Structures of cell wall arabinosyltransferases with the anti-tuberculosis drug ethambutol. *Science* 2020;368:1211–1219.
